# Simultaneous CK2/TNIK/DYRK1 inhibition by 108600 suppresses triple negative breast cancer stem cells and chemotherapy-resistant disease

**DOI:** 10.1038/s41467-021-24878-z

**Published:** 2021-08-03

**Authors:** Katsutoshi Sato, Amol A. Padgaonkar, Stacey J. Baker, Stephen C. Cosenza, Olga Rechkoblit, D. R. C. Venkata Subbaiah, Josep Domingo-Domenech, Alison Bartkowski, Elisa R. Port, Aneel K. Aggarwal, M. V. Ramana Reddy, Hanna Y. Irie, E. Premkumar Reddy

**Affiliations:** 1 Division of Hematology and Medical Oncology, Department of Medicine, Icahn School of Medicine at Mount Sinai, New York, NY USA; 2 Department of Oncological Sciences, Icahn School of Medicine at Mount Sinai, New York, NY USA; 3 Department of Pharmacological Sciences, Icahn School of Medicine at Mount Sinai, New York, NY USA; 4 Medical Oncology and Cancer Biology, Thomas Jefferson University, Philadelphia, PA USA; 5 Department of Surgery, Mount Sinai Hospital, New York, NY USA

**Keywords:** Breast cancer, Breast cancer

## Abstract

Triple negative breast cancer (TNBC) remains challenging because of heterogeneous responses to chemotherapy. Incomplete response is associated with a greater risk of metastatic progression. Therefore, treatments that target chemotherapy-resistant TNBC and enhance chemosensitivity would improve outcomes for these high-risk patients. Breast cancer stem cell-like cells (BCSCs) have been proposed to represent a chemotherapy-resistant subpopulation responsible for tumor initiation, progression and metastases. Targeting this population could lead to improved TNBC disease control. Here, we describe a novel multi-kinase inhibitor, 108600, that targets the TNBC BCSC population. 108600 treatment suppresses growth, colony and mammosphere forming capacity of BCSCs and induces G2M arrest and apoptosis of TNBC cells. In vivo, 108600 treatment of mice bearing triple negative tumors results in the induction of apoptosis and overcomes chemotherapy resistance. Finally, treatment with 108600 and chemotherapy suppresses growth of pre-established TNBC metastases, providing additional support for the clinical translation of this agent to clinical trials.

## Abstract (web summary)

Finding therapeutic strategies for aggressive triple negative breast cancer (TNBC) is an important challenge in clinical practice. Here, the authors identify a multi-kinases inhibitor with antitumor activity and able overcome chemotherapy resistance of TNBC in vivo.

## Introduction

Triple negative breast cancers (TNBCs), defined as tumors that lack expression of estrogen receptor (ER), progesterone receptor (PR), and human epidermal growth factor receptor-2 (HER2) represent ~15% of diagnosed breast cancers^1^. While some patients do well with standard of care multi-modality therapy that includes chemotherapy, many patients have suboptimal responses. Chemotherapy resistance is a negative prognostic factor with poorer survival in TNBC^2,3^ and is thought to be due to the presence of breast cancer stem cells (BCSCs), a subpopulation of cells that resides within the tumor^4,5^. These cells are relatively resistant to standard chemotherapeutic agents, and have the ability to renew, grow as mammospheres in vitro and initiate tumor development in mouse models. At a molecular level, BCSCs are generally defined as CD44^high^CD24^low^EpCAM^+^ and/or have high levels of aldehyde dehydrogenase-1 (ALDH1^high^) enzyme activity^6,7^. Several studies have shown that sorted human breast cancer CD44^high^/CD24^low^EpCAM^+^ cells have increased tumorigenic potential in mouse xenograft assays^6^. Like normal stem cells, BCSCs divide asymmetrically, generating additional BCSCs that display the same characteristics as the parental cell (CD44^high^/CD24^low^) as well as differentiate into daughter cells (CD44^low^CD24^low^).

The metastatic and invasive properties of TNBC are attributed to CD44, which is thought to mediate invasion via urokinase-type plasminogen activators (uPAs)^8,9^. Furthermore, ALDH1, a BCSC marker, is statistically correlated with decreased survival and poor prognosis^10,11^. Together, these studies strongly suggest that targeting CD44^high^/CD24^low^/ALDH1^+^ cells is likely to result in a successful therapeutic strategy. Identification of new proteins and pathways in tumor cells from RNAi screens has presented new opportunities for drug development, both in terms of using RNAi as a therapeutic agent and identifying new targets. However, a common finding from these screens is that many genes are required for the survival of tumor cells, making it difficult to select a single target for drug development^12,13^. This complexity has led to a recent and renewed interest in targeted, polypharmacological approaches for developing multi-kinase inhibitors with low toxicity profiles^14^.

In an attempt to understand the molecular basis of TNBC growth and to develop a targeted therapy for this disease, we designed an approach where we combined the identification of “oncogenic kinases” with drug discovery. Because growth of most tumors is dictated by the activation of kinase cascades, we designed a strategy to identify possible oncogenic kinases upon which a breast cancer stem cell might be dependent. This was made possible because of our library of ~4000 novel compounds, which consists of ~2000 ATP-mimetics which function as kinase inhibitors. We used this group of compounds to determine if any of these agents can induce the death of CD44^high^/CD24^low^ BCSCs purified from TNBC cells. This approach allowed two important aspects of drug discovery to occur simultaneously: the identification of new therapeutic targets and the development of novel pharmaceuticals that can inhibit these targets. This strategy identified 108600, which shows remarkable efficacy against triple negative breast tumor cells, including the BCSC population, with minimal toxicity towards normal cells. Our studies presented here show that 108600 is a potent inhibitor of TNBC growth in vitro and in vivo, including chemotherapy-resistant and metastatic disease. Our data support clinical translation of 108600 as a complement to standard chemotherapy for high risk, aggressive TNBC to improve patient outcomes.

## Results

### Derivation of a small molecule inhibitor of TNBC growth

**Fig. 1:**
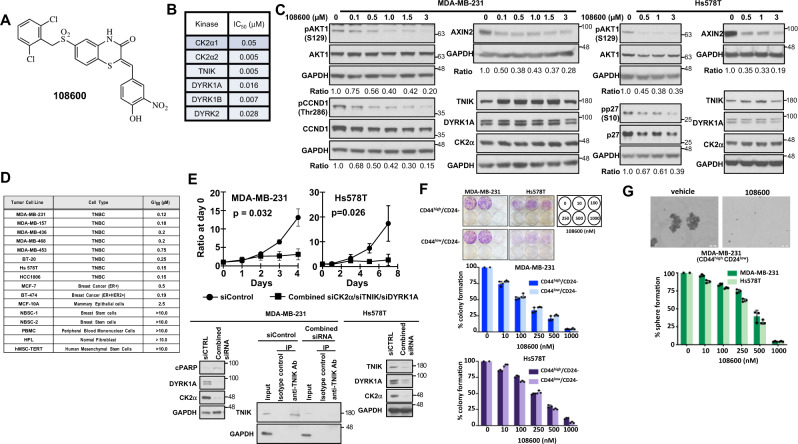
108600 suppresses the growth of TNBC stem cells. **A** Chemical structure of 108600. **B** IC_50_ values for inhibition of 108600 target kinases. **C** 108600 inhibits phosphorylation of CK2α, DYRK1A and TNIK substrates. Lysates derived from MDA-MB-231 and Hs578T cells treated for 24 h with 108600 at the indicated concentrations were probed for expression of phospho-AKT1 (Ser129), phospho-CYCLIN D1 (Thr286), and AXIN2. The blots are representative of three independent experiments. Samples derive from the same experiment and the blots processed in parallel. **D** GI_50_ values for growth inhibition of breast cancer and normal cells treated with 108600. **E** Simultaneous siRNA-mediated knockdown of CK2α, DYRK1A, and TNIK suppresses proliferation of MDA-MB-231 and Hs578T cells. siRNA-transfected cells were counted on the indicated days. All data represent mean values ± standard deviation obtained from three independent biological replicates. The statistical difference between the target and nontarget siRNA treated groups was tested using two-way ANOVA (two-sided). Knockdown was confirmed by western blot analysis, which was performed three times. Samples derive from the same experiment and the blots processed in parallel. **F** 108600 treatment suppresses colony and **G** mammosphere formation of CD44^high^/CD24^low^ stem cell fraction of MDA-MB-231 and Hs578T cells. For colony assays, CD44^high^/CD24^low^ cells were purified by FACS, treated with the indicated concentrations of 108600 for 72 h, washed and allowed to grow for 7–14 days in drug-free medium. Cells were fixed with 4% paraformaldehyde in PBS, stained with crystal violet solution and colonies quantified microscopically. All data points represent mean value ± standard deviation. Scale bars = 50 µm. Values in **F** and G represent the mean percentage of colony and sphere formation ±SD, respectively, relative to that of DMSO, which was set to 100%, in three independent experiments. Source data are provided as a source data file.

In this study, we used phenotypic screening of TNBCs as an approach to identify the nature of signal transduction pathways activated in these cells and simultaneously identify compounds that can target these pathways. To accomplish this goal, we used four TNBC cell lines MDA-MB-231, MDA-MB-468, Hs578T, and BT-20 as well as the CD44^high^/CD24^low^ BCSC purified populations derived from these lines to screen a compound library synthesized in our laboratory to identify compounds that induce apoptosis of these cells. This strategy identified 108600 (Fig. 1A), which showed remarkable toxicity towards the bulk and BCSC populations of these cell lines, with little toxicity to normal cells. This compound was assayed against a panel of 285 functional kinases by Reaction Biology Corporation (RBC), which revealed that 108600 is a multi-kinase inhibitor and inhibits CK2α1, α2, DYRK1A, 1B, and DYRK2 and TNIK at low nanomolar concentrations (Fig. 1B and Supplementary Fig 1).

To confirm that 108600 inhibited endogenous CK2, DYRK, and TNIK activity in a dose-dependent manner in TNBC cells, we evaluated the effect of 108600 treatment on established substrates of these kinases in MDA-MB231 and Hs578T cells, as well as organoids derived from a primary TNBC with intrinsic resistance to anthracycline and taxane-based chemotherapy (CTG1883). CK2 has been shown to phosphorylate AKT1 at serine (S) 129, which promotes a hyperactivated state of AKT1^15,16^. Additionally, threonine (Thr) 286 of CYCLIN D1 and serine (Ser)10 of p27 are known phosphorylation sites for DYRK1A^17^. Treatment with 108600 led to decreased phosphorylation at these sites (Fig. 1C and supplementary Fig 2), as well as decreased levels of autophosphorylated DYRK1A/B (Supplementary Fig 3), indicating that 108600 treatment suppresses endogenous CK2α and DYRK1 activities in TNBC cells. We also examined the effect of 108600 on the activity of TNIK, which regulates Wnt signaling as a core component of the β-catenin transcriptional complex. AXIN2 expression, which is induced by activated Wnt/TCF/LEF^18–20^ was used as readout of TNIK activity, and as predicted, was suppressed in 108600-treated cells (Fig. 1C).

### 108600 inhibits viability of TNBC CD44^high^/CD24^low^ breast cancer stem cells

We next evaluated the effect of 108600 treatment on growth of an expanded panel of breast cancer cell lines, as well as six normal and nontransformed cells. The kinases targeted by 108600 are robustly expressed in most TNBC cell lines, with relatively lower expression in normal cells (fibroblasts, immortalized human-mesenchymal stem cells) (Supplementary Fig. 4). 108600 potently inhibited the viability of all triple negative and other breast tumor cell lines with little or no toxicity against normal cells, including normal mammary epithelial cell lines derived from two different donors (Fig. 1D). The growth suppression of TNBC cells (MDA-MB231 and Hs578T) observed with 108600 treatment was phenocopied by simultaneous transfection of cells with three siRNA pools targeting CK2α, DYRK1A, and TNIK (Fig. 1E). Transfection of MDA-MB-231 or Hs578T cells with siRNA pools individually targeting CK2α, DYRK1A, or TNIK had little or moderate effects on growth, supporting the need for simultaneous multi-kinase targeting (Supplementary Fig. 5). Individual siRNAs targeting CK2α, DYRK1A, or TNIK suppressed phosphorylation of their respective, reported substrates, thereby phenocopying the effects of 108600 treatment and further validating that these kinases are indeed targeted by 108600 (Supplementary Fig 6).

To specifically examine the effect of 108600 treatment on the BCSC population, we purified CD44^high^/CD24^low^ cells from two TNBC cell lines (MDA-MB-231 and Hs578T) by FACS (according to schema in Supplementary Fig 7) and examined the effects of 108600 treatment on colony and mammosphere forming capabilities of the CD44^high^/CD24^low^ fraction. 108600 potently inhibited colony growth of the CD44^high^/CD24^low^ population, as well as CD44^low^/CD24^low^ cells (Fig. 1F). We also examined the effects of 108600 on patient-derived TNBC cells in 2D and 3D cultures using commercially available CD44^high^/CD24^low^/ALDH+ breast cancer stem cells derived from a TNBC patient. 108600 potently inhibited mammosphere formation by these cells in in vitro 2D and 3D cultures (Supplementary Fig. 8), which is in agreement with the data obtained from sorted populations isolated from established TNBC cell lines (Fig. 1G).

### Structure of CK2α1 in complex with 108600

**Fig. 2:**
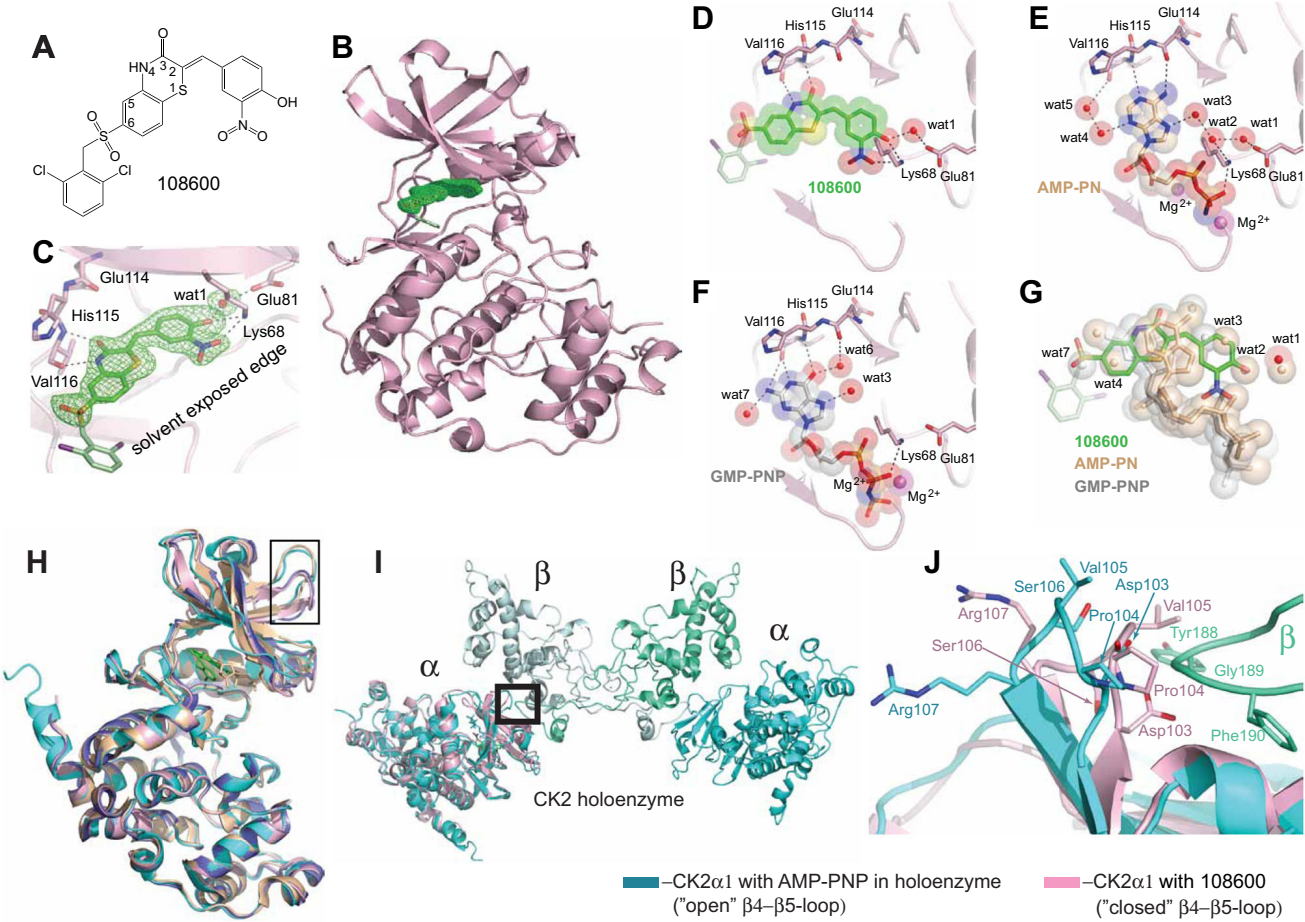
Crystal structure of 108600 in complex with CK2α. **A** Chemical structure of 108600. **B** Overall structure of the CK2α1-108600 complex. CK2α1 protein (pink) is shown in cartoon. 108600 is shown in sticks. A simulated annealing Fo-Fc omit electron density map for 108600 (green) is contoured at 3.0 σ-level at 1.80 Å resolution. **C** Zoom-in view of 108600 interactions with CK2α1. The water molecule (wat1) is shown as a red sphere. The electron density for the 2,6-dichlorobenzyl ring of the drug is disordered due to the conformational flexibility of this aromatic moiety in the solvent-exposed region of the active site; **D** A close-up view of 108600 interactions with CK2α1 depicting a surface view of the drug. **E**, **F** AMP-PNP (PDB ID: 3NSZ)^21^ (the γ-phosphate of AMP-PNP is hydrolyzed due to the acidic crystallization conditions to yield AMP-PN) and GMP-PNP (PDB ID: 1DAY)^22^ accommodations within the active site of CK2α1. The water-mediated ligand–protein interactions are also shown. **G** 108600, AMP-PN, and GMP-PNP in surface representation in the active site of CK2α1. Significantly, the drug mimics not only the shape and electrostatics of AMP-PN and GMP-PNP, but also their hydration patterns. **H**–**J** 108600 induces change in the conformation of the β4-β5 loop, which interacts with the regulatory CK2β subunit in the holoenzyme complex. **H** Superposition of CK2α1 structures with AMP-PNP alone (PDB ID: 3NSZ, beige) and integrated in the holoenzyme complex with CK2β regulatory subunit dimer (PDB ID: 1JWH, cyan)^23^, CK2α1 in the absence of ligand (PDB ID: 1NAZ, blue), and CK2α1 with 108600 (pink). Black frame highlights the β4-β5 loop. **I** CK2 kinase holoenzyme (PDB ID: 1JWH) and CK2α1-108600 complex. The holoenzyme consists of the two CK2α1 subunits (cyan) and the two CK2β regulatory subunits (green). CK2α1 subunit of the holoenzyme contains AMP-PNP. The CK2α1 β4-β5 loop region and its interaction with the loop of the CK2β subunit are highlighted by a thick black frame. **J** The “closed” conformation of the β4–β5 loop in the CK2α1/108600 complex (pink) appears to interfere with interactions with the loop of the CK2β subunit (green–blue) in the CK2 holoenzyme^2^.

To gain an understanding of the mechanisms by which 108600 inhibits CK2 kinases, we expressed, purified, crystallized, and determined the structure of the human CK2α1 catalytic domain in complex with 108600 (Supplementary Fig. 11). The structure was solved by molecular replacement and refined to 1.80 Å resolution. The structure of CK2α1 has the familiar bilobal architecture of a protein kinase, composed of N-terminal lobe followed by a larger C-terminal lobe (Fig. 2B). 108600 binds in the cleft between the two domains, where ATP would normally be accommodated (Figs. 2A, B and Supplementary Fig. 12) and adopts a conformation where the NO_2_ group and the aromatic ring are coplanar (Fig. 2C). The density for 2,6-dichlorobenzyl ring of the drug is disordered due to the conformational flexibility of this aromatic moiety in the solvent-exposed region of the active site (Fig. 2A–D).

Previous work has shown that CK2 is able to use both ATP and GTP as phosphoryl donors with similar efficiencies. The crystal structure of the human CK2α1 in complex with the ATP nonhydrolysable analogue AMP-PNP has been determined at a 1.3 Å resolution (Fig. 2E)^21^. The CK2α1 (Zea mays) complex structure with GMP-PNP resolved at 2.2 Å showed that GMP-PNP and water molecules mimic AMP-PNP in the active site of protein kinase CK2^22^. Our studies show that the O3 and N4 of 108600 take the positions of the O6 and N1 atoms of GMP-PNP, respectively, and maintain the same interactions with the backbone atoms of His115 and Val116 (Fig. 2D, F). Moreover, the NO_2_ group of the drug is near the position that is occupied by the Pα group of AMP-PNP or GMP-PNP and establishes a hydrogen bond with the side chain of Lys68 (Fig. 2D, E, F). In addition, the central benzothiazinone and aromatic rings are involved in numerous hydrophobic contacts with residues such as Leu45, Val66, Ile95, Phe113, and Ile174 that line the ATP/GTP binding cavity (Supplementary Fig. 12). The carbonyl group of the thiazone ring interacts with the main chain NH group of His115 and the NH proton of the ring establishes a hydrogen bond with the main chain carbonyl group of Val116. Importantly, a water molecule (wat1) in the drug complex forms hydrogen bonds with the OH group of the drug and the side chain of Glu81 (Fig. 2D). Wat1 is also observed in the complex with AMP-PNP (Fig. 2E). Moreover, the drug covers the area where wat2, wat3, and wat4 are bound in the AMP-PNP/CK2α1 complex (Fig. 2E) and wat7 in the GMP-PNP/ CK2^α^1 complex (Fig. 2F). To compare the binding modes of 108600 and AMP-PNP/GMP-PNP, the coordinates for all complexes were superimposed (Fig. 2G). Significantly, the drug mimics not only the shape and electrostatics of AMP-PNP/GMP-PNP, but also their hydration patterns in the CK2α1 active site pocket (Fig. 2D, E, F, G).

### 108600 induces a conformational change in CK2^α^1, which is not conducive to holoenzyme formation

The CK2 holoenzyme consists of two catalytic subunits (CK2^α^1 or CK2^α^2), which interact with a CK2β dimer forming a transient complex^23^. Interestingly, the CK2 holoenzyme has been shown to bind substrates and activate them in a kinase-independent manner^24^. CK2α1-AMP-PNP either alone or integrated into the holoenzyme has the loop connecting β4 and β5 sheets in the “open” conformation (Fig. 2H) conductive to integration with CK2β subunit (Fig. 2I, J). In the absence of AMP-PNP, the β4–β5 loop is in the “closed” conformation (Fig. 2H). Intriguingly, in the presence of 108600, the β4–β5 loop adopts the “closed” conformation that is obstructive to holoenzyme formation (Fig. 2H, I, J). Hence, 108600 binding to CK2α1 appears to induce a conformational change, which represents a state that is not conducive to holoenzyme formation (Fig. 2I, J). 108600 may thus inhibit CK2 as a both a competitive inhibitor of ATP/GTP and as an allosteric inhibitor that weakens the interaction between C2Kα and C2Kβ in vivo.

### 108600 inhibits tubulin polymerization and causes abnormal mitosis

**Fig. 3:**
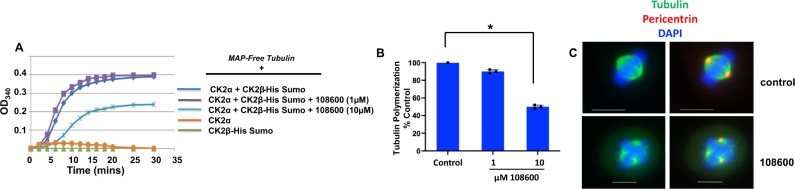
108600 suppresses tubulin polymerization. **A** Recombinant CK2α or CK2β or CK2α + CK2β holoenzyme preparations were pretreated with either DMSO or 108600 for 5 min and the mixture was added to MAP-free tubulins in buffer containing 1 mM GTP. The reaction mixtures were transferred to a heated spectrophotometer and tubulin polymerization was evaluated by monitoring absorbance at 340 nm over a period of 40 min. Representative results from experiment are shown; **B** Graphical representation of % inhibition of polymerization by 108600. Values represent the mean percentage of tubulin polymerization ±SD relative to that of DMSO, which was set to 100%, in three independent experiments Statistical differences were tested using one sample *t*- and Wilcoxin tests (two tailed). The *p* value of 0.0346 is indicated by an asterisk and is regarded as statistically significant.; **C** Exponentially growing HeLa cells were treated with vehicle (DMSO) or 108600 for 12 h, fixed and stained with α-tubulin and pericentrin antibodies and DAPI. Representative images of two technical replicates using confocal microscopy show abnormal mitotic spindle formation induced by treatment with 108600. Scale bars = 10 µm and 100 µm for control and 108600-treated cells, respectively. Source data are provided as a source data file.

Small interfering RNAs directed against CK2 α and β subunits exhibit mitotic abnormalities ranging from defects in centrosome duplication, anaphase spindle elongation, and chromosome mis-segregation^25^. The CK2 holoenzyme is an established microtubule associated protein (MAP) that aids in tubulin polymerization and regulates microtubule cytoskeletal reorganization^26,27^. Since 108600 induces a conformational change in the CK2α subunit by potentially interfering with its ability to bind to the β-subunit, we examined the effects 108600 on CK2-holoenzyme-mediated tubulin dynamics in vitro and assessed its effects on mitosis. We adapted and employed a published protocol^27^, which measures CK2-holoenzyme-mediated de novo tubulin polymerization. MAP-free tubulin preparations were incubated with recombinant purified CK2α, CK2β-His-Sumo or pre-assembled (CK2α-CK2β) holoenzyme, respectively, and the extent of tubulin polymerization was measured over time. We did not observe any tubulin polymerization in the presence of individual CK2 subunits (CK2α or CK2β), even after a prolonged incubation (Fig. 3A, B). However, addition of the CK2 holoenzyme to tubulin resulted in enhanced polymerization, which is in agreement with published observations that the CK2 holoenzyme promotes tubulin polymerization. When we pretreated the CK2 holoenzyme with 108600, we observed a decrease in CK2-holoenzyme-mediated tubulin polymerization (Fig. 3A, B). These effects suggest that 108600 inhibits CK2-holoenzyme formation and hence, tubulin dynamics.

To study the mitotic phenotype induced by 108600, cells were grown on collagen coated glass coverslips and treated with either DMSO or 108600 overnight and stained for α-tubulin and the centrosomal marker, pericentrin. While DMSO-treated mitotic cells displayed normal bipolar spindle architecture, the cells treated with 108600 formed multipolar spindles and multiple centrosomes (Fig. 3C). These effects are similar to those observed with CK2 subunit inhibition reported by Bettencourt-Dias et al^25^, further supporting the fact that 108600 inhibits CK2-dependent functions.

### 108600 treatment induces cell cycle arrest and apoptosis of TNBC cells

**Fig. 4:**
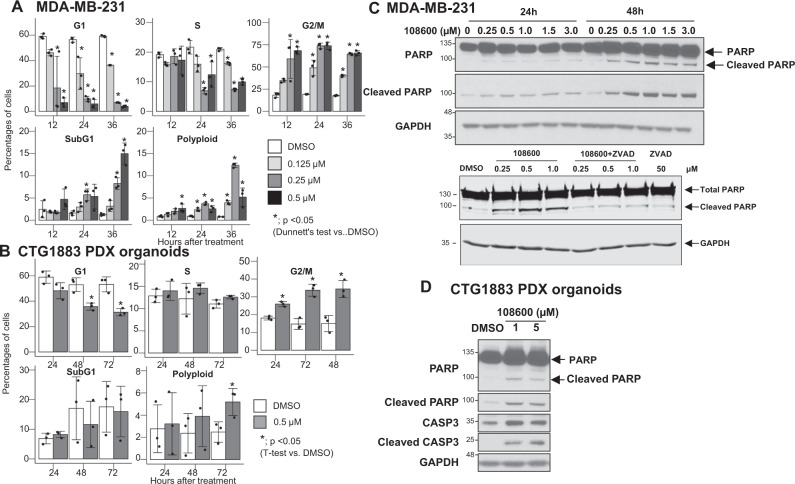
108600 induces G2/M arrest and apoptosis of TNBC cells and organoids. **A** MDA-MB-231 cells were treated with the indicated concentrations of 108600, stained with propidium iodide (PI) and analyzed by flow cytometry. Statistical differences were calculated using Dunnett’s test (two-sided). A *p* value of less than 0.05 is indicated by an asterisk and is regarded as statistically significant. **B** CTG1883 TNBC organoids were treated with 108600 (0.5 µM), stained with PI and analyzed by flow cytometry. All data represent mean values ± standard deviation obtained from three independent biological replicates. Statistical differences were tested using *t*-test (two-sided). A p value of less than 0.05 is indicated by an asterisk and is regarded as statistically significant. **C** MDA-MB-231 cells were treated with 108600 for 24 or 48 h. Lysates derived from these cells were subjected to western blot analysis and probed for the expression of total or cleaved PARP (top), which is inhibited by a 2 h pretreatment with the ZVAD (50 µM) caspase inhibitor (bottom). Samples derive from the same experiment and the blots processed in parallel. The blots are representative of three independent experiments. **D** CTG1883 organoids were treated with 108600 for 48 h. Lysates from these cells were probed for the expression of total or cleaved PARP and caspase 3. All westerns were performed on the same gel. The blot is representative of three independent experiments. Source data are provided as a source data file.

Given the potent inhibition of TNBC cell growth observed with 108600 treatment (Fig. 1) and its effects on tubulin polymerization and mitosis (Fig. 3), we assessed cell cycle progression of triple negative MDA-MB-231 cells and CTG1883 TNBC organoids treated with increasing concentrations of 108600 for varying periods of time. Treatment with 108600 resulted in a potent, dose- and time-dependent G2/M arrest (Fig. 4A, B and Supplementary Fig. 13) as assessed by flow cytometric analysis (Supplementary Fig 10). The percentages of cells in the G2/M phase were clearly increased with 108600 treatment, while the fraction of cells in the G1 or S phases concomitantly decreased. Notably, 108600 treatment increased the percentage of cells in the sub-G1 fraction, indicating apoptotic cells, and increased the percentage of polyploid or aneuploid cells. Interestingly, when we performed these studies with normal proliferating cells such as hMSC-hTERT (immortalized human-mesenchymal stem) or MCF-10A (human mammary epithelial) cells, which express very low levels of CK2, we observed minimal or no mitotic arrest in cells treated with 1 µM 108600 (Supplementary Fig 13). The majority of these cells remained in the G1 phase, with a modest increase in the G2/M population. Taken together, these results suggest that 108600 does not induce mitotic arrest of normal cells, which might explain its selective toxicity against tumor cells.

These observations strongly suggest that treatment of TNBC with 108600 leads to prolonged G2/M arrest and eventually induces cell death. To more specifically determine whether 108600 treatment induces apoptosis, we examined levels of cleaved PARP and cleaved Caspase 3 in cells treated with increasing doses of 108600 for 24–48 h. Increased PARP cleavage, which was blocked by the addition of the ZVAD caspase inhibitor, was detected in both MDA-MB-231 cells and TNBC PDX organoids (Fig. 4C, D, supporting the induction of apoptosis by 108600.

### Chemotherapy-resistant TNBCs are sensitive to 108600 treatment

**Fig. 5:**
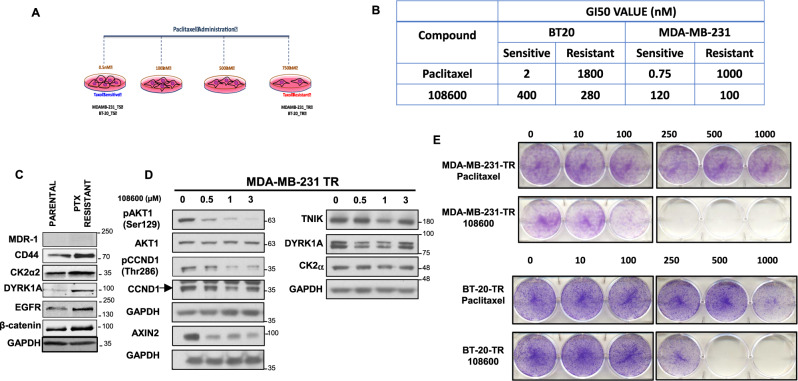
108600 suppresses the growth of chemotherapy-resistant TNBC cells. **A** Schematic diagram showing the strategy used for the generation of paclitaxel-resistant TNBC cell lines. MDA-MB-231 or BT-20 cells were grown in increasing concentrations of paclitaxel and GI_50_ values were determined after every passage. Each subsequent passage was maintained in the highest possible concentration of paclitaxel-containing medium. Selection was completed when resistance reached > 200-fold; **B** GI_50_ values for paclitaxel and 108600 for parental, paclitaxel-sensitive (TS) and paclitaxel-resistant (TR) MDA-MB-231 and BT-20 cells using CellTiter Blue in conjunction with clonogenic assays. **C** Expression of MDR-1, CD44, CK2, DYRK1A, EGFR, β-Catenin in paclitaxel-sensitive and resistant MDA-MB-231 cells as determined by western blot analysis. GAPDH serves as a loading control. Images are representative of two independent experiments. Samples derive from the same experiment and the blots processed in parallel. **D** Paclitaxel-resistant MDA-MB-231 cells were treated with increasing concentrations of 108600 for 24 h. Lysates derived from these cells were subjected to western blot analysis using the indicated antibodies. The blot is representative of three independent experiments. Samples derive from the same experiment and the blots processed in parallel. **E** Paclitaxel-resistant MDA-MB-231 cells were treated with increasing concentrations of paclitaxel or 108600 for 72 h, washed and allowed to grow for 7 days in drug-free growth medium. Colonies were stained with crystal violet. Source data are provided as a source data file.

Patients with TNBC are often treated with anthracycline and taxane-based chemotherapy as standard of care. However, as response to these chemotherapies is heterogenous, we determined whether 108600 treatment would also be effective in suppressing viability and inducing apoptosis of chemotherapy-resistant TNBC cells in vitro. We generated paclitaxel-resistant variants of the MDA-MB-231 and BT-20 cell lines by exposing them to increasing concentrations of paclitaxel (PTX). Resistance was validated by cell viability, colony formation and apoptosis assays. After continuous exposure and passaging in PTX-containing medium, we obtained MDA-MB-231 and BT-20 cells (MDA-MB-231-TR and BT-20-TR), which exhibited greater than 250-fold resistance to PTX as compared to the parental, PTX-sensitive (TS) cell lines (Fig. 5B).

We investigated whether the CD44^high^CD24^-/low^ stem-cell-like population was increased in the PTX-resistant cell lines, similar to those of patients who are refractory to taxane therapy^28^.Interestingly, western blot analysis of parental and MDA-MB-231-TR cells showed an increase in the level of CD44 expression relative to the parental cell line, which might reflect an increase in the BCSC population (Fig. 5C). In addition, target kinases of 108600, such as DYRK1A, were also upregulated in the PTX-R variant (Fig. 5C). To determine whether 108600 inhibits signaling in the context of PTX-resistance, PTX-R MDA-MB-231 cells were treated with increasing concentrations of 108600 for 24 h and the phosphorylation status of CK2, DYRK, and TNIK substrates analyzed by western blot analysis. Fig. 5D and Supplementary fig. 3 show that treatment with 108600 resulted in an inhibition of both pAKT1 Ser129 and pCYCLIN D1 Thr286 phosphorylation, as well as a reduction in the level of AXIN2 expression and DYRK1A/B autophosphorylation, confirming inhibition of these target kinases (Fig. 5D). 108600 also potently inhibited growth and colony formation of both PTX-sensitive and PTX-resistant MDA-MB-231 and BT-20 cell lines with the same efficiency (Fig. 5E), suggesting that it effectively overrides PTX-resistance in vitro.

### 108600 inhibits the growth of TNBC in vivo

**Fig. 6:**
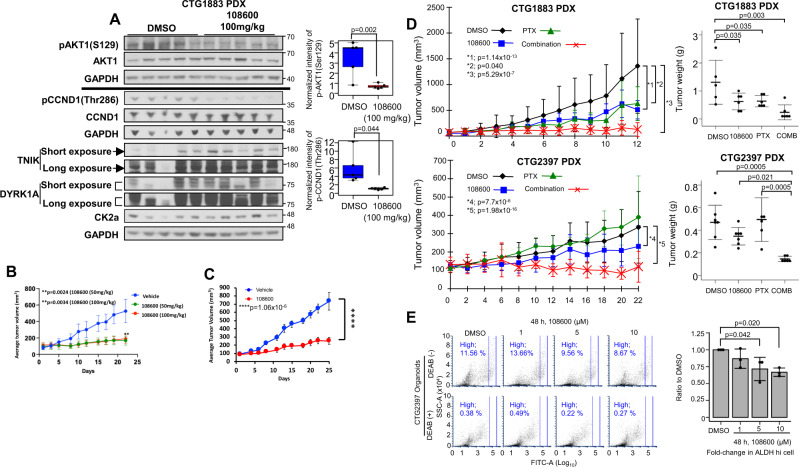
108600 suppresses growth of TNBC cell line and PDX tumor grafts. **A** 108600 inhibits phosphorylation of CK2α and DYRK1A substrates in vivo. NSG mice bearing CTG1883 PDX tumor grafts were treated with 108600 (100 mg/kg) or DMSO for 5 days (*n* = 5). Box plots show levels of phospho-AKT1^Ser129^ and phospho-CYCLIN D1^Thr 286^ normalized to those of their respective total proteins and GAPDH. Variances were tested using F-tests (two-sided). *P* values were calculated using a *t*-test (two-sided). Samples derive from the same experiment and the blots processed in parallel. **B** Nude mice bearing MDA-MB-231 xenografts were treated with vehicle or 108600 (50 mg/kg or 100 mg/kg) for 21 days. Tumor volumes were measured on the indicated days. (*n* = 7 for vehicle- and 108600 (100 mg/kg)-treated animals; *n* = 6 for 108600 (50 mg/kg)-treated animals). Statistical differences were assessed by two-way ANOVA (two-sided). Data represent mean ± SEM. **C** 108600 suppresses growth of paclitaxel-sensitive PDX tumors. TM00098 tumor-bearing NSG mice were randomly assigned to groups and treated with vehicle (*n* = 4) or 108600 (100 mg/kg q2D) (*n* = 5). Error bars represent mean ± SEM. Statistical differences were assessed by two-way ANOVA (two-sided). **D** Chemotherapy-resistant PDX growth suppression by 108600. PDX models were treated with DMSO (*n* = 5 for CTG1883; *n* = 7 for CTG2397), paclitaxel (10 mg/kg, *n* = 6 for CTG1883; *n* = 5 for CTG2397), 108600 (100 mg/kg, *n* = 6 for CTG1883; *n* = 7 for CTG2397) or a combination of paclitaxel and 108600 (*n* = 6 for CTG1883; *n* = 6 for CTG2397). Paclitaxel and 108600 were administered q4d and q2d, respectively. Statistical differences were assessed by two-way ANOVA (two-sided). Weights of tumors harvested at study endpoints are shown. Data represent mean ± SD. Statistical differences were assessed using a *t*-test with false discovery rate correction calculated by the Benjamini–Hochberg method. Experiments were performed twice with each model. **E** 108600 decreased the ALDH^high^ fraction in CTG2397 organoids. ALDH activity was measured in organoids treated with 108600 for 48 h. DEAB-treated organoids served as negative controls. Mean values ± SD from three independent experiments are shown. Statistical differences between the ALDH^high^ fractions were tested using one-way ANOVA (two-sided) and Dunnett’s test (two-sided). *p* values of less than 0.05 were considered statistically significant. Source data are provided as a source data file.

Before determining the efficacy of 108600 against TNBC growth in vivo, we first established a dosing regimen based on toxicity data and suppression of 108600 target kinase activities. We performed a pilot study whereby CTG1883 tumor-bearing NSG mice were treated with vehicle or 108600 (100 mg/kg) for a 5-day period. Whole-cell extracts derived from the tumors were subjected to western blot analysis using antibodies directed against pAKT1 Ser129, pCYCLIN D1 Thr286, and C-MYC as readouts for CK2α, DYRK1A, and TNIK kinase inhibition, respectively. (C-MYC, like AXIN2, is activated by Wnt/TCF/LEF signaling^19,20^). Fig. 6A and supplementary fig 14 show that the levels of pAKT1 Ser129, pCYCLIN D1 Thr286 and C-MYC were decreased in the tumors isolated from 108600-treated animals, suggesting that loss of AKT phosphorylation and/or that of CYCLIN D1 as well as MYC expression might serve as relevant biomarkers in vivo. Based on the results of this experiment, administration of 108600 on a q2D schedule at a dose of 100 mg/kg was chosen for subsequent studies in tumor-bearing mouse models.

To determine the efficacy of 108600 against TNBC growth in vivo, MDA-MB-231 cells were injected subcutaneously into the mammary fat pads of female athymic (NCr-nu/nu) mice and the tumors allowed to grow to a size of 100–150 mm^3^. The mice were then randomly grouped and treated every other day with 50 mg/kg or 100 mg/kg of 108600 or an equal volume of placebo. Tumor measurements and body weights were measured on the indicated days and at the end of the study, the tumors (or site of inoculation) harvested and subjected to further analysis. Fig. 6B shows that 108600 is a potent inhibitor of TNBC tumor growth as judged by the reduction in tumor volumes in both 108600-treated cohorts (Fig. 6B). The study had to be terminated at the end of 22 days due to the size of tumors in the control group, which began to undergo necrosis. Measurement of body weights and gross visual examination of drug-treated animals did not show any detectable toxicity (Supplementary Fig. 15).

To expand the clinical relevance of 108600 as a therapeutic for TNBC, we also examined the effect of 108600 on the growth of an invasive ductal carcinoma TNBC PDX (TM00098), which, like the parental MDA-MB-231 cell line, is sensitive to taxane therapy. Tumor-bearing nod/scid/gamma (NSG) mice were randomly assigned to two groups and treated with 108600 (100 mg/kg) or an equal volume of placebo as described above. The results presented in Fig. 6C shows that treatment with 108600 suppressed the growth of this tumor in vivo without adverse effects on the animals’ body weights (Supplementary Fig. 15)

### 108600 suppresses aggressive TNBC PDX growth in combination with chemotherapy

To further evaluate the efficacy of 108600 in suppressing growth of chemotherapy-resistant TNBC in vivo, we utilized clinically annotated PDX models of drug-resistant TNBC that we have been developing as part of an ongoing co-clinical study. We prioritized models based on relative chemotherapy resistance of the original patient’s cancer from which the PDX model was derived, as well as levels of expression of 108600 targets (CK2, DYRK, and TNIK kinases; supplementary fig 16). After establishing the dose of 108600 that is sufficient to suppress established substrates of 108600 target kinases (Fig. 6A and supplementary fig 16), we propagated two PDX models, CTG1883 and CTG2397, for use in efficacy studies based on robust expression of 108600 targets. As previously stated, CTG1883 is derived from a primary TNBC with intrinsic resistance to anthracycline and taxane-based chemotherapy, while CTG2397 is derived from a metastatic TNBC that recurred despite anthracycline and taxane-based chemotherapy. For these studies, mice bearing PDX tumor grafts were treated with either vehicle control, PTX (10 mg/kg q4D), 108600 (100 mg/kg q2D) or a combination of PTX/108600, and tumor growth was monitored over a period of 12–24 days. While paclitaxel had a moderate or minimal inhibitory effect on tumor growth in the CTG1883 and CTG2397 models, respectively, 108600 as a single agent significantly suppressed tumor growth in both models. However, the combination of 108600 and PTX resulted in a near complete suppression of tumor growth in both chemotherapy-resistant PDX models, as assessed by caliper measurement and endpoint tumor weight (Fig. 6D) without adversely affecting the animals’ body weights (Supplementary Fig. 15). Combined treatment with 108600/PTX synergistically inhibited tumor growth, as supported by an interaction effect on rates of change in tumor growth in this treatment group when compared to those in the 108600 and PTX treated cohorts (Supplementary Fig 17).

We next evaluated the effect of 108600 on the stem-cell-like population in these PDX tumors by assessing ALDH activity in organoids derived from these models (Fig. 6E and Supplementary Fig. 18). 108600 treatment suppressed the frequency of the ALDH-high fraction in CTG1883 organoid cultures in a dose-dependent manner. These results collectively support 108600’s efficacy against chemotherapy-resistant TNBC is achieved due to suppression of the BCSC population, thereby enhancing sensitivity to chemotherapy.

### 108600 suppresses growth of pre-established TNBC metastases

**Fig. 7:**
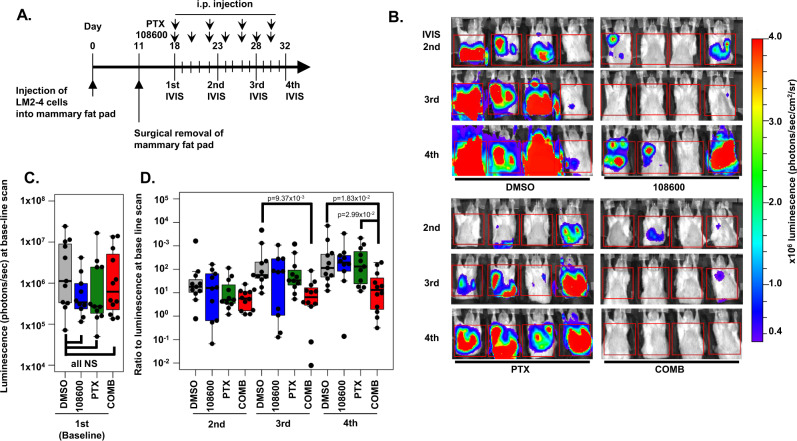
108600 and paclitaxel synergize to inhibit established TNBC metastases. MDA-MB-231/LM2-4 mCherry cells were used to generate tumors in the mammary fat pads of 7-week-old NSG mice. Surgery to remove the primary tumor was performed and all mice were imaged by IVIS for the presence of metastases. **A** Schema of the experiment. Mice with metastases were randomized into four treatment groups (*n* = 10–12 per group) and metastatic disease was monitored by serial IVIS imaging. **B** Serial IVIS imaging of mice in the four treatment groups. **C** Total luminescence in the region-of-interest (lung) window for the four groups at time of treatment randomization (1st IVIS), showing no statistically significant difference in lung metastases burden (Dunnett’s test, two-sided). **D** The ratio of luminescence intensity compared to baseline luminescence (**C**) for each treatment group at serial timepoints indicated in the schema. Ratios were calculated at each IVIS imaging. *P* values were calculated with Dunnett’s (two-sided) and Tukey’s tests (two-sided) in comparison to the value for the DMSO-treated group. Each data point represents data obtained for an individual animal, with the top and bottom of the box corresponding to the 75th and 25th percentiles, respectively. Data points that are higher or lower than 1.5 times that of the interquartile range within the box were regarded as outliers.

The results from the above in vivo studies encouraged us to investigate the efficacy of 108600 treatment against TNBC that has already metastasized. This is particularly important as most novel therapeutics are first evaluated in the context of advanced, metastatic disease that has previously been exposed to prior chemotherapy. For these studies, MDA-MB231 LM2-4/mCherry Luc cells (LM2-4/mCherry), a luciferase and mCherry-expressing derivative of MDA-MB-231 that readily disseminates from the primary site to form metastases in lung and liver, was used for modeling metastatic breast cancer^29^. LM2-4/mCherry Luc cells were implanted into the mammary fat pads of NSG mice and the primary tumors removed prior to evidence of metastasis (11 days post-implantation). Serial in vivo imaging (IVIS) was performed to monitor the development of metastases and once metastases were detected in the majority of animals (primarily in the lung), mice with metastases were randomized into four treatment groups: (1) DMSO vehicle control; (2) 108600 (100 mg/kg q2D); (3) PTX (10 mg/kg q4D); or (4) a combination of 108600 and PTX. A total of 10–12 mice/treatment group were serially imaged for metastases on days 18, 23, 28, and 32 by IVIS (Fig. 7A, B), using the same acquisition settings for all treatment groups. Acquisition timing after luciferin injection (15 min), exposure time (5 min), lung gates (3.3 cm horizontal × 3.3 cm vertical), were kept constant for all treatment groups and timepoints to enable comparisons. At baseline, there was no significant difference in metastatic disease burden among the four groups (Fig. 7C). We also determined the ratio of luciferase signal from lung metastases at different treatment timepoints (2nd, 3rd, 4th IVIS) relative to the signal obtained at time of randomization (1st IVIS) (Fig. 7D). While treatment with paclitaxel had little or no effect on metastatic progression, treatment with 108600 alone had an early suppressive effect on metastatic burden but the effect was only partially sustained. However, combined treatment with 108600 and PTX significantly and stably suppressed growth of already established lung metastases. (Fig. 7D). These promising results show that 108600 synergizes with chemotherapy to suppress TNBC metastatic growth, and support clinical translation of 108600/chemotherapy combination treatment for stage 4 TNBC.

## Discussion

Resistance to chemotherapy remains a critical problem facing patients diagnosed with TNBC, especially since suboptimal response to chemotherapy is a strong predictor of long-term poor prognosis. Multiple mechanisms have been proposed to contribute to chemotherapy resistance, among which is the presence of a subpopulation of cells within the tumor that has stem-cell-like properties with tumor initiation and self-renewal capacity^30,31^. This population, which is characterized by CD44^high^/CD24^low^ expression^32,33^ as well as by high ALDH activity^34^, is associated with chemotherapy resistance and worse prognosis for multiple tumor types, and may contribute to tumor progression and metastasis^7,35–38^. Therapeutically targeting this population could enhance chemosensitivity and improve outcomes for patients. Here, we have identified a novel compound, 108600, identified in a screen for novel therapeutics that suppress the BCSC-like population. 108600 synergizes with chemotherapy to inhibit growth of aggressive TNBC that is resistant to standard of care chemotherapy.

The efficacy of 108600 lies in its potent simultaneous inhibition of multiple signaling pathways that are active in CSCs and are required for their maintenance and/or propagation. 108600’s primary targets, CK2, DYRK, and TNIK kinases, collectively activate signaling molecules such as STAT3^39,40^, Wnt/β-catenin^41–43^, PI3K/AKT^15,16^, hedgehog^44,45^, and Notch1^46^. These proteins have been reported to be active in the CSC population, are required for stem cell maintenance^47^ and contribute to drug resistance and metastasis^30,31^. CK2, while ubiquitously expressed, appears to be especially critical for growth and survival of cancer cells^48–50^, with its activation being associated with aggressive tumor behavior and higher levels correlating with poorer prognoses across multiple tumor types^29,51–55^. CK2 promotes the activation of AKT^15,16^ and β-catenin^42^ by direct phosphorylation, and NF-κβ signaling by phosphorylation and degradation of IκBα^56–59^. Among the relevant substrates of CK2 that could impact chemotherapy sensitivity are ABC proteins (P-gp/MDR and MRP1) that are pumps responsible for efflux of chemotherapeutic agents^60^, and XRCC1/XRCC4 which regulate DNA single and double-stranded break repair^61^. Inhibition of CK2 kinase activity or its expression has been shown to inhibit growth and induced apoptosis of multiple cancer cell types in vitro^62–65^ and suppress the stem-like tumor initiating cells in glioblastomas^66,67^.

In addition to CK2, other primary targets of 108600 play key roles in the behavior of cancer stem cells. Class I DYRK kinases have diverse functions in cell proliferation and differentiation by regulating the degradation of key cell cycle regulators such as CYCLIN D1 and p27^KIP1^ via phosphorylation^17,68^. These kinases also modulate gene expression through direct effects on transcription factors and chromatin modification. For example, DYRK1A activates transcription factors critical for stem cell maintenance such as GLI1, a transcriptional effector of Hedgehog signaling, and STAT3^69,70^. TNIK, another 108600 target, activates Wnt/β-catenin signaling as a component of the β-catenin/TCF4 transcriptional complex^19,71^, in addition to activating JNK and NF-κB^72^. Importantly, this kinase also plays a key role in the maintenance of tumor stem cells in vivo^20^.

Multiple mechanisms are responsible for the growth inhibition and apoptosis induced by 108600 treatment. Our crystallographic studies show that these effects are likely mediated, in part, by direct inhibition of CK2-holoenzyme complex formation (Fig. 2). At the cellular level, CK2 localizes to the mitotic apparatus and regulates levels of core cell cycle proteins^49,73^. In agreement with these studies, inhibition of tubulin polymerization, chromosome mis-segregation and potent G2/M arrest observed with short-term 108600 treatment lead to the induction apoptosis (Figs. 3, 4 and supplementary fig. 12)^74^. 108600-treated cells are likely sensitized to apoptotic triggers due to inhibition of multiple CK2/DYRK/TNIK-dependent survival signaling pathways (AKT, NF-kB, STAT), activation of caspases and direct suppression of anti-apoptotic Bcl-2 family members, such as Bcl-x_L_ and survivin^43,58,75^. Finally, suppression of cells with ALDH-high, CSC-like characteristics, as we observed in organoid cultures derived from a chemotherapy-resistant TNBC PDX model treated with 108600 (Fig. 6), might be critical for sensitizing malignant cells to standard of care chemotherapy and eliminating a drug-resistant population.

Small kinase inhibitors that individually target CK2, DYRK1, or TNIK kinase are in various stages of preclinical development. Treatment with NC-1, an oral inhibitor of TNIK, was reported to suppress the ALDH-positive colon cancer CSC population, as well as growth of tumor xenografts in preclinical models^76^. An orally available CK2 inhibitor (CX-4945) has already been evaluated in Phase 1 clinical trials for solid tumors. While this agent was found to be generally well tolerated, only partial responses were observed^77,78^. These findings support the need for simultaneous inhibition of multiple signaling pathways to overcome drug resistance in advanced cancers. Such findings are also consistent with our results showing that individual downregulation of CK2α, DYRK1A, or TNIK had negligible effects in inhibiting TNBC growth, whereas simultaneous downregulation of all three kinases was required to phenocopy the effects of 108600 treatment (Fig. 1 and supplementary fig. 4). Despite potent multi-kinase inhibition, 108600 treatment does not induce cell death of normal cells (Fig. 1) and toxicities were not observed in our in vivo studies using mouse models.

Our cell biological studies show that 108600 is extremely potent in suppressing the growth of both CD44^high^/CD24^low^ and CD44^low^/CD24^low^ TNBCs (Fig. 1). Moreover, 108600 induces apoptosis of paclitaxel-resistant TNBCs in vitro (Fig. 5). We also examined the efficacy of 108600 using MDA-MB-231 xenografts as well as two PDX models of TNBC that are resistant to paclitaxel. 108600 acts synergistically with paclitaxel in both PDX models (Fig. 6), even when the tumors were non-responsive to paclitaxel as a single agent. This observation is particularly significant since chemotherapy resistance remains a critical problem facing patients diagnosed with TNBC. Another major issue encountered with TNBC patients is the highly metastatic nature of this disease, which is the primary cause of patient mortality. Our studies with LM2-4 cells, a highly metastatic variant of the MDA-MB-231 cell line, show that 108600 is an effective suppressor of established metastases when administered in combination with paclitaxel (Fig. 7 and supplementary fig. 17). This property of 108600 may prove to be highly advantageous to patients with advanced TNBC whose cancers are resistant to chemotherapy.

In addition to direct effects on the tumor cell compartment, it is possible that 108600 induces favorable changes in the immune microenvironment of TNBC tumors that promote effective antitumor immunity. Recently, it was reported that CK2 inhibition suppressed the differentiation of myeloid-derived suppressor cells and tumor associated macrophages and synergized with immune checkpoint inhibitors to suppress tumor growth^79^. Therefore, 108600 could have effects on the immune microenvironment that also contribute to its potent antitumor effects, raising the possibility that it might synergize with immunotherapies.

In summary, we have identified a potent multi-kinase inhibitor of CSCs that suppresses TNBC growth and overcomes resistance to chemotherapy. 108600’s efficacy against established metastatic disease and its favorable toxicity profile make it a promising therapeutic for clinical translation. Effective inhibition of chemotherapy-resistant TNBC, particularly the CSC-subpopulation, could lead to dramatic improvements in morbidity and mortality associated with TNBC.

## Methods

### Cell lines

Established cell lines were purchased from ATCC. LM2-4/mCherry Luc cells (LM2-4/mCherry) were a gift from Dr. Robert Kerbel (Sunnybrook) and were maintained in Roswell Park Memorial Institute (RPMI) medium (ThermoFisher Scientific) containing 5% FBS. MDA-MB-231, BT-20 and hMSC-hTERT cell lines were maintained in DMEM supplemented with 10% FBS and 1× penicillin-streptomycin. Hs578T cells were cultured in DMEM supplemented with 10% FBS and 10 µg/ml insulin (Sigma–Aldrich). HFLs were cultured in Ham’s F12 nutrient mixture (ThermoFisher Scientific) supplanted with 10% FBS and 1× penicillin-streptomycin. All cell lines were maintained at 37 °C under humidified conditions and 5% CO_2_.

PDX organoid cultures were maintained in F12/DMEM (GE healthcare, Chicago, IL) containing 5% FBS (ThermoFisher Scientific), 10 mM HEPES (ThermoFisher Scientific), 1 μg/ml bovine serum albumin (BSA) (Sigma–Aldrich, St. Louis, MO), 1× insulin-transferrin-selenium (ThermoFisher Scientific), 0.5 µg/ml hydrocortisone (Sigma–Aldrich), 2.5 µg/ml Fungizone (ThermoFisher Scientific), 50 µg/ml Gentamicin (ThermoFisher Scientific), and 5 μM ROCK inhibitor (Y27632) (Selleck Chemicals, Houston, TX).

### Animals

Female nu/nu mice were purchased from Charles River Laboratories or Taconic. Female NOD.Cg-Prkdc^scid^Il2rg^tm1Wjl^/SzJ (NSG) and TNBC PDX (TM00098)-bearing NSG mice were purchased from the Jackson Laboratory. Mice used in all studies were 8–12 weeks of age at the start of the study. Animals were housed in climate-controlled barrier conditions with automated light/dark cycles. All animal experiments were performed under protocols approved by the Icahn School of Medicine at Mount Sinai’s Institutional Animal Care and Use Committee according to federal and institutional guidelines and regulations.

### Patient samples

Samples from patients treated at the Mount Sinai Hospital were collected for PDX generation and expansion. Written informed consent was obtained for all subjects on Icahn School of Medicine at Mount Sinai (ISMMS) Institutional Review Board (IRB)–approved protocol (HSM# 14-00330) prior to the procedure at which the specimen was obtained. The studies were conducted in accordance with the Belmont Report and U.S. Common Rule and approved by the ISMMS IRB.

### Reagents

#### Antibodies

Primary antibodies were purchased from Cell Signaling Technologies: phospho-AKT1 (Ser129) (#13461), DYRK1A (#2771), DYRK1B (#2703), AKT1 (#2967), phospho-CYCLIN D1 (Thr286) (#3300), CYCLIN D1 (#2926), p27 (#3698), AXIN2 (#2151), TNIK (#32712), cleaved PARP (#9541 and #32563), PARP (#9532 and #9542), cleaved Caspase 3 (#9661), CD44 (#3507), MDR-1 (#13342), c-Myc (#5605); Abcam: CK2α1 (ab76040), phospho-p27 (Ser10) (ab62364), pericentrin (ab4448); Santa Cruz Biotechnology: GAPDH (sc-365062), TNIK (sc-136103), CK2α2 (sc-9030), EGFR (sc-53274); BD Biosciences: PE CD44 (561858, 1:500), FITC CD24 (560992, 1:200); Invitrogen: ß-catenin (AH00462); phospho-DYRK (PA5-64574). Secondary antibodies were purchased from Cell Signaling Technologies (horse radish peroxidase [HRP] conjugated anti-rabbit IgG [#7074]) or LICOR Biosciences (IRDye^®^ 680 Goat anti-Mouse IgG (H + L) #926-32220, IRDye^®^ 800CW Goat anti-Rabbit IgG (H + L), #926-32211, IRDye^®^ 680RD Goat anti-Mouse IgG #926–68070, IRDye^®^ 680RD Goat anti-Mouse IgG #926–32210). Primary and secondary antibodies for western blot analysis were used at 1:1,000 and 1:10,000 dilutions, respectively. Antibodies were used at a 1:200 dilution in immunofluorescence staining.

#### Chemicals

Carbobenzoxy-valyl-alanyl-aspartyl-[O-methyl]-fluoromethylketone (Z-VAD-FMK) was purchased from Cell Signaling Technology and dissolved in dimethyl sulfoxide (DMSO) prior to use. Paclitaxel (Sigma–Aldrich) was dissolved into 1:1 mixture of ethanol and Cremophore EL (GE healthcare) for in vivo experiments. Four volumes of phosphate buffer saline without Ca^2+^ and Mg^2+^ PBS(−) was added to the paclitaxel:cremophore mixture immediately before every injection. 108600 was dissolved in DMSO immediately prior to each injection.

### Western blot analysis

Protein concentrations of whole-cell lysates were measured using the Lowry or bicinchoninic acid (BCA) assay. Lysates were resolved by SDS-PAGE, transferred onto an Immobilon PVDF or nitrocellulose membrane and incubated in primary antibody overnight at 4 °C. After secondary antibody incubation, the membranes were developed using ECL Western Blotting Substrate (ThermoFisher Scientific or MiliporeSigma) or an Odyssey CLx imaging system (LICOR Biosciences).

For in vivo tumor lysates, tumors were harvested 3 h after the final treatment and immediately snap frozen in liquid nitrogen. Frozen tumors were ground using mortar and pestle and immediately lysed at 4 °C using radioimmunoprecipitation assay (RIPA) buffer (Cell Signaling, Denver, MA). The lysates were centrifuged at 22,000 × *g* for 20 min at 4 °C, and the clarified supernatant was collected for subsequent western blot analysis.

### Immunoprecipitation

Whole-cell lysates were harvested with RIPA buffer (Cell signaling) and the concentrations measured using the BCA assay. One milligram of whole-cell lysate was incubated with anti-TNIK antibody (Santa Cruz Biotechnology Inc., sc-136103) or its isotype control (Santa Cruz Biotechnology Inc., sc-3877) for 2 h at 4 °C. The lysates were then incubated with protein A/G-conjugated agarose (Santa Cruz Biotech., sc-2003) for 1 h at 4 °C, and centrifuged at 1000 × *g* for 5 min. to collect the precipitates. The precipitates were then washed four times with RIPA buffer and denatured using 20 µL of 1× SDS sample buffer. An identical concentration of whole-cell lysate was used as an input control. Western blot analysis using anti-TNIK (Cell Signaling Technologies, #32712) antibodies were performed to measure TNIK expression.

### CK2α1 protein expression, purification, and co-crystallization with 108600

The CK2α1 catalytic domain was expressed and purified as described previously^80^. Briefly, a construct consisting of residues 3–339 encompassing the CK2α1 catalytic domain was expressed in *E. coli* as His6-SUMO (small ubiquitin-like modifier) N-terminally tagged fusion protein. The His6-SUMO tag was removed by cleavage with Ulp1 protease after Ni-NTA affinity chromatography and the protein was further purified by Ni-NTA, heparin, and size-exclusion gel-filtration column chromatography (Supplementary Fig. 9). The crystals of CK2a1-108600 complex were obtained by the vapor diffusion method at 20 °C and grew as thin plates. To set up a crystallization drop, 1 μl of 10 mg/ml CK2α1 protein was mixed with 1 μl of the reservoir solution containing 100 mM Tris-HCl pH 7.5, 200 mM ammonium sulfate and 21% PEG5000 on a cover slide. 0.2 μl of 10 mM 108600 solution was then added to the drop. 108600 was dissolved in 25% Cremophor EL, 24.5% ethanol, 40% PEG 400, 5 mM Tris-HCL pH 8.0. The use of Cremophor EL-PEG 400-buffer mixture for 108600 solution preparation was critical for co-crystallization since 108600 dissolved in DMSO resulted in heavy precipitation in the crystallization drop and the absence of crystal growth. The CK2α1-108600 complex crystals diffracted to high 1.80 Å resolution at 23ID synchrotron beamline at the Argonne National Laboratory. The structure was solved by molecular replacement with the CK2α1 catalytic domain (PDB ID: 3OWJ)^81^ as a search model. The final model was refined to 1.80 Å resolution and R_work_/R_free_ 17.5/22.1 (Supplementary Fig. 9E).

### Tubulin polymerization assays

Microtubules were assembled in vitro from purified Microtubule Associated Protein (MAP)-free tubulin (Cytoskeleton Cat#TL238c) at 2 mg/ml in manufacturer-supplied PEM buffer supplemented with 1 mm GTP at 35 °C, and the turbidity of the solutions was monitored at 340 nm. DMSO or 108600 at varying concentrations was preincubated with recombinant purified CK2α1 (30 ng/ml) for 30 min and an equal concentration of recombinant CK2β was then added to the mixture. This mixture was then added to the MAP-free tubulin to initiate CK2-holoenzyme-dependent polymerization. The rate of tubulin polymerization was measured using densitometry at 2-minute intervals.

### siRNA transfection

siRNA transfections were performed using Oligofectamine (ThermoFisher Scientific, Inc.). siGENOME SMART pools (Horizon Discovery) for CSNK2A1 (M-003475-03-0005), DYRK1A (M-00485-01-005), and TNIK (M-004542-03-0005), and non-targeting control siRNAs (D-001210-05-50) were transfected according to the manufacturer’s protocol. Briefly, the culture medium was changed to Opti-MEM (ThermoFisher Scientific, Inc.) prior to transfection, and the mixture of Oligofectamine and 30 nM of each siRNA pool were added to the cells. Media containing 3× serum was added to the cells after a 5 h incubation at 37 °C. The cells were harvested 96 h post-transfection.

### Flow cytometry

#### Cell cycle analysis

Organoids were washed and harvested with PBS (−) and Tryp-LE (ThermoFisher Scientific), and then fixed with ice-cold 95% ethanol for a minimum of 24 h. The cells were then washed twice with PBS(−) and stained with PI/RNase staining buffer (Becton Dickinson, Franklin Lakes, NJ) for 15 min. at room temperature. Flow cytometric analysis was performed using an LSRII Fortessa (Becton Dickinson) and the data analyzed using FCS Express 6.0 (De Novo Software, Glendale, CA) or FlowJo™ v9 (BD Biosciences).

#### Aldefluor assay

ALDEFLOUR™ Kit (Stemcell Technologies, Vancouver, Canada) was used according to manufacturer’s instructions. Organoids were washed twice with PBS(−) and then incubated with digestion buffer, (1 mg/ml Collagenase IV, 100 units/ml hyalronidase, and 100 µg/ml DNase I in PBS(−)), with shaking at 200 rpm. for 15 min at 37 °C. Organoids were incubated with Aldeflour solution at 37 °C for 60 min. DEAB (Stemcell Technologies) treatment was used as a negative control to set the gating for the ALDH^high^ fraction.

#### Isolation of BCSC populations

Single-cell suspensions were prepared in phenol red-free RPMI supplemented with 2% heat-inactivated FBS. Defined numbers of cells were stained on ice for 1 h, washed, and then subjected to flow cytometric analysis. Antibodies used were as follows: anti-CD44 PE (BD Biosciences 561858) and anti-CD24 FITC (BD Biosciences 560992). DAPI was used at a concentration of 10 ng/ml as a measurement of viability. Flow cytometric sorting was performed by the Flow Cytometry Shared Resource Facility at the Icahn School of Medicine at Mount Sinai using a FACSAria II (BD Biosciences). Data were acquired using FACSDiva^TM^ v8.0 and analyzed using FCS Express 6.0 (De Novo Software, Glendale, CA) or FlowJo™ v9 (BD Biosciences).

### Immunofluorescence and confocal microscopy

Exponentially growing cells were seeded at a density of at 0.1 × 105 cells/ml in 100 mm dishes containing sterilized glass microscope coverslips and allowed to transfix overnight. The next day, cells were treated with DMSO or 108600 for 12 h. Coverslips were gently washed with PBS, fixed with 4% paraformaldehyde for 15 min and washed three times with PBS for 5 mins. Cells were then permeabilized using 0.1% Triton X-100 in PBS for 15 min at room temperature. After another PBS wash, coverslips were incubated in blocking buffer (1% BSA in PBST) for 1 h at 37 °C. The cells were incubated with anti-α-tubulin-FITC (Sigma–Aldrich # F2168) and anti-pericentrin (Abcam, ab4448) antibodies diluted 1:200 in blocking buffer for 1 h at 37 °C. The coverslips were again washed three times with PBS and stained with Texas red goat anti-rabbit secondary antibody (BD Biosciences, T2767). Coverslips were washed again with PBS and incubated in 1 μg/mL DAPI solution (Southernbiotech 0100-20) for 5 min before being washed two times with PBS. Finally, coverslips were mounted onto glass slides using ProLong^®^ Gold Antifade Reagent (ThermoFisher Scientific, P36930) and dried overnight in the dark before being imaged.

### In vitro kinase assays

In vitro kinase assays were performed as previously described by us^82^. Briefly, 10 ng of recombinant protein (ThermoFisher Scientific) was diluted in kinase buffer (20 mM HEPES [pH 7.5], 10 mM MgCl_2_, 1 mM EGTA, 0.01% Brij35, 0.02 mg/ml BSA, 0.1 mM Na_3_VO_4_, 2 mM DTT, 1% DMSO and incubated with the indicated concentrations of 108600 at room temperature for 30 min. Kinase reactions were initiated by the addition of 1 μg substrate and mixture of 10 µM ATP (Sigma–Aldrich) and 100 µCi ^33^P-γ-ATP (PerkinElmer). The reactions were incubated for 2 h at 25 °C and subsequently spotted onto P81 ion exchange filter paper. The filters were then thoroughly washed in 0.75% phosphoric acid to remove unbound phosphate and the level of substrate radioactivity quantitated using a scintillation counter. Values were plotted as a function of log drug concentration and IC_50_ values determined by plotting sigmoidal non-linear regression curves with a variable slope. Peptide substrates used were as follows: CK2α and CK2α2: RRRDDDSDDD; TNIK: RLGDKYKTLRQIRQ; DYRK1: RRRFRPASPLRGPPK.

### Viability assays and GI_50_ value determination

2.5 × 10^3^ cells were seeded into each well of a black, clear-bottom 96-well plate and treated with 108600 24 h post-plating. Fluorescence was measured by excitation at 579 nm and an emission at 594 nm after a 96 h incubation using CellTiter-Blue reagent (Promega). GI_50_ values were calculated by normalizing fluorescence values to those of DMSO-treated controls after background subtraction.

### In vivo tumor efficacy studies

For MDA-MB-231 xenografts, 1 × 10^7^ cells in 0.2 ml of sterile PBS were subcutaneously injected into the mammary fat pads of Ncr-nu/nu mice. PDX tumor grafts were propagated as follows: 4–5 mm frozen tumor fragments were subcutaneously transplanted into the right flank of NSG mice. Approximately 1 month after primary transplantation, the growing tumors were harvested, cut into 5 mm tumor fragments, and retransplanted into cohorts of NSG mice. When tumor grafts reached ~50–100 mm^3^ in size, treatment was initiated with intraperitoneal injections of paclitaxel or 108600 injected every 4 or 2 days, respectively. DMSO was used as control. Body weights and tumor volumes were measured by calipers every 2 or 3 days and volume estimated using the formula: ½ (width^2^ × length). Where indicated, preengrafted, PDX-bearing NSG mice (TM00098) purchased from the Jackson Laboratory were used for efficacy studies that followed the above dosing and monitoring protocols.

### In vivo imaging (IVIS)

5 × 10^5^ LM2-4/mCherry cells were injected into the right inguinal mammary fat pad of NSG mice. Eleven days after injection, the primary tumor and the mammary fat pad were surgically removed. NSG mice were anesthetized using inflowing isoflurane (2–2.5%) in 2–2.5 L/min oxygen, and placed into the imaging chamber, and background luminescence was acquired. 10 µL/g mouse body weight of luciferin (PerkinElmer Inc., Waltham, MA) was injected intraperitoneally. One minute after the injection of luciferin, luminescence was acquired to check for presence of metastasis or residual tumor. To block luminescence from any residual primary tumor, the area around the inguinal mammary fat pad was covered with black aluminum foil and clay. Luciferin signals were acquired 5, 15, and 25 min. after luciferin injection. The luminescence intensity at 15 min. was used for the quantitative analysis. To analyze the lung metastasis, a 3.3 × 3.3 cm window was set as region of interest (ROI) prior to measuring radiance.

Mice with metastases were selected based on the luminescence signal at the first IVIS imaging following primary tumor removal, and each mouse was randomly assigned to DMSO, 108600, paclitaxel or 108600/paclitaxel combination treatment groups. DMSO and 108600 were intraperitoneally injected every 2 days and paclitaxel was injected every 4 days for 2 weeks. The luminescence intensity at the first IVIS imaging was used as a baseline intensity for each mouse, and the ratio of luminescence between each IVIS imaging and baseline was used for comparison. Living Image (version 4.7.2, PerkinElmer Inc.) was used for acquisition and analysis of luminescence intensity.

### Preparation of organoid cultures

Frozen small tumor fragments in Cellbanker 1 (Nippon Zenyaku Kogyo) at −80 °C were used for making organoids. After thawing, the fragments were cut, minced and incubated in digestion buffer at 37 °C with shaking. After digestion, the cell suspension was filtered (100 μm) and centrifuged. Pellets were collected and resuspended in human breast epithelial cell (HBEC) medium (5% FBS, 10 mM HEPES, BSA, 0.5 µg/ml hydrocortisone, 50 µg/ml gentamycin, 2.5 µg/ml Fungizone, 1× insulin-transferrin-selenium, and 5 μM ROCK inhibitor (Y27632) (Selleck Chemicals) in DMEM/F12^83^. Ficoll density gradient centrifugation was performed and the live cell fraction was collected. After washing with PBS, cells were seeded on nonadherent 24-well plate. Nonviable cells were removed by density gradient centrifugation 3 days post-plating.

### Colony-formation assays

Sorted cells were plated into six-well plates at 750 cells/well and allowed to adhere overnight. 108600 was then added at varying concentrations for a 72 h period. Drug-containing medium was replaced with fresh medium and the cells were incubated for an additional 8–10 days with media changes made every 3 days. Cells were fixed in methanol (−20 °C) and stained with 5% crystal violet. Colonies were counted using ImageJ v1.5.

### Mammosphere-formation assays

TNBC cell lines, sorted BCSCs from cell lines and patient-derived human breast cancer stem cells (XLC479, Creative Bioarray) were cultured in suspension in serum free DMEM/F12 1:1 supplemented with EGF (20 ng/ml, BD Biosciences), B27 (1:50, Invitrogen), 0.4% bovine serum albumin (Sigma–Aldrich), and 4 μg/ml insulin (Sigma–Aldrich) on Costar ultralow attachment multi-well plates (Corning Cat#3471). Cells were grown in DMSO or 108600 (200 nM) for 72 h. Drug-containing medium was replaced with fresh medium and the cells were further incubated for 1–2 weeks to induce mammosphere formation.

### Reporting summary

Further information on research design is available in the Nature Research Reporting Summary linked to this article.

## Supplementary information

Supplementary Information

Reporting Summary

## Data Availability

Atomic coordinates and structure factors have been deposited in the Protein Data Bank (PDB) under accession code 7L1X. Source data are provided with this paper.

## Source data

Source Data

## Materials availability

Request for materials generated in this study should be directed to the corresponding authors and will be made available upon execution of a Material Transfer Agreement.
