# Supplementary material for: Simultaneous CK2/TNIK/DYRK1 inhibition by 108600 suppresses triple negative breast cancer stem cells and chemotherapy-resistant disease
Source: Nat Commun. 2021 Aug 3;12:4671. doi: 10.1038/s41467-021-24878-z (PMC8333338; doi:10.1038/s41467-021-24878-z)
Supplement: Supplementary file 1 — Supplementary Information [file 41467_2021_24878_MOESM1_ESM.pdf]

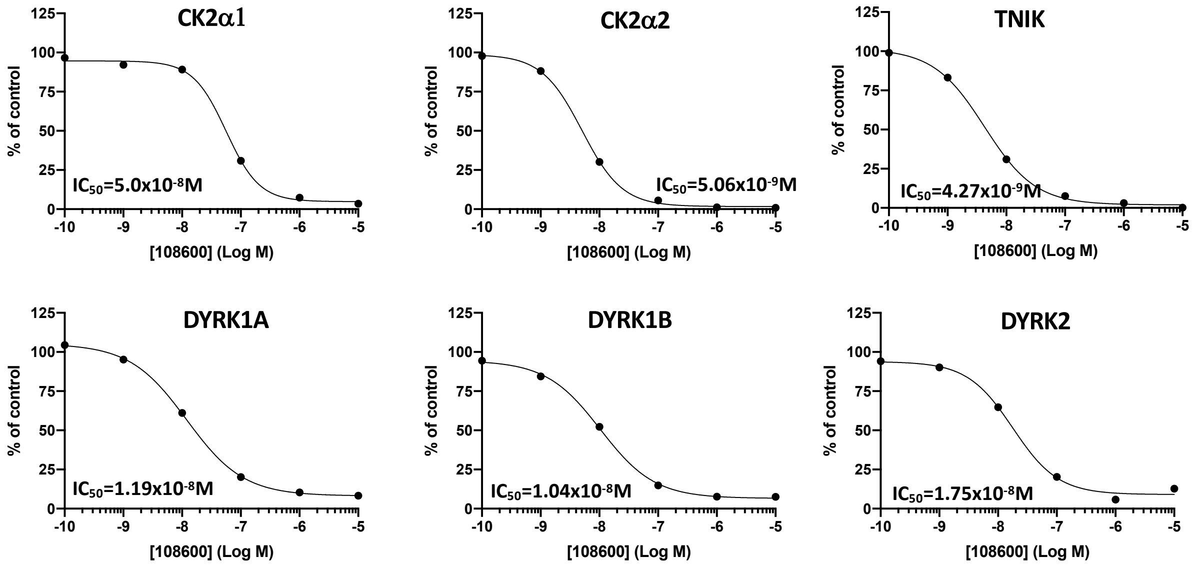

Supplementary Figure 1. Kinase inhibition of 108600 targets *in vitro*. Recombinant proteins were incubated with the indicated concentrations of 108600 and subjected to *in vitro* kinase assays. Values obtained were plotted as a function of log drug concentration. IC_{50} values were determined by plotting sigmoidal non-linear regression curves with a variable slope.

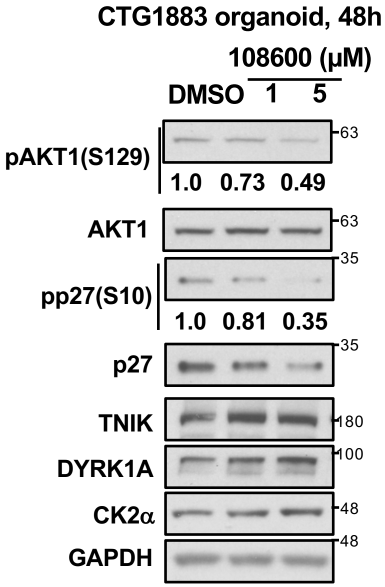

Supplementary Figure 2. 108600 inhibits target kinases in TNBC PDX organoids. CTG1883 PDX organoids were treated with 108600 and phosphorylation status of substrates of CK2 and DYRK1 were analyzed by Western blot analysis. The numbers indicate relative ratios of phosphorylated substrates (normalized) in 108600- versus DMSO-treated organoids. The blots are representative of three independent experiments. Source data are provided as a source data file.

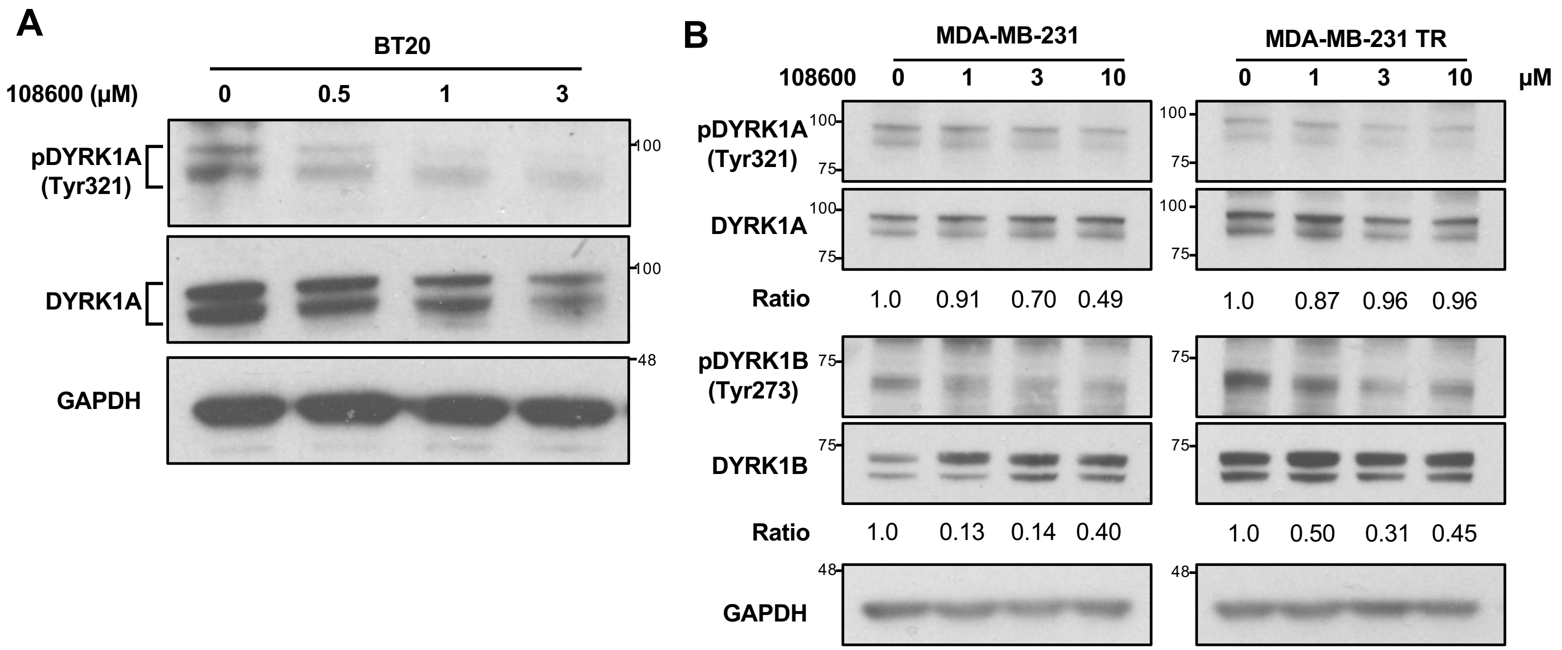

Supplementary Figure 3. 108600 treatment suppresses autophosphorylation of DYRK1. (A) BT-20, (B) MDA-MD-231 and MDA-MB-231 paclitaxel resistant (TR) cells were treated with increasing concentrations of 108600 for 24 hours, lysed and the lysates subjected to Western blot analysis using an antibody directed against phosphorylated DYRK1A/B (Tyr321/Tyr273). The numbers indicate relative ratios of phosphorylated substrates (normalized) in 108600- versus DMSO-treated cells. The blots are representative of three independent experiments. Source data are provided as a source data file.

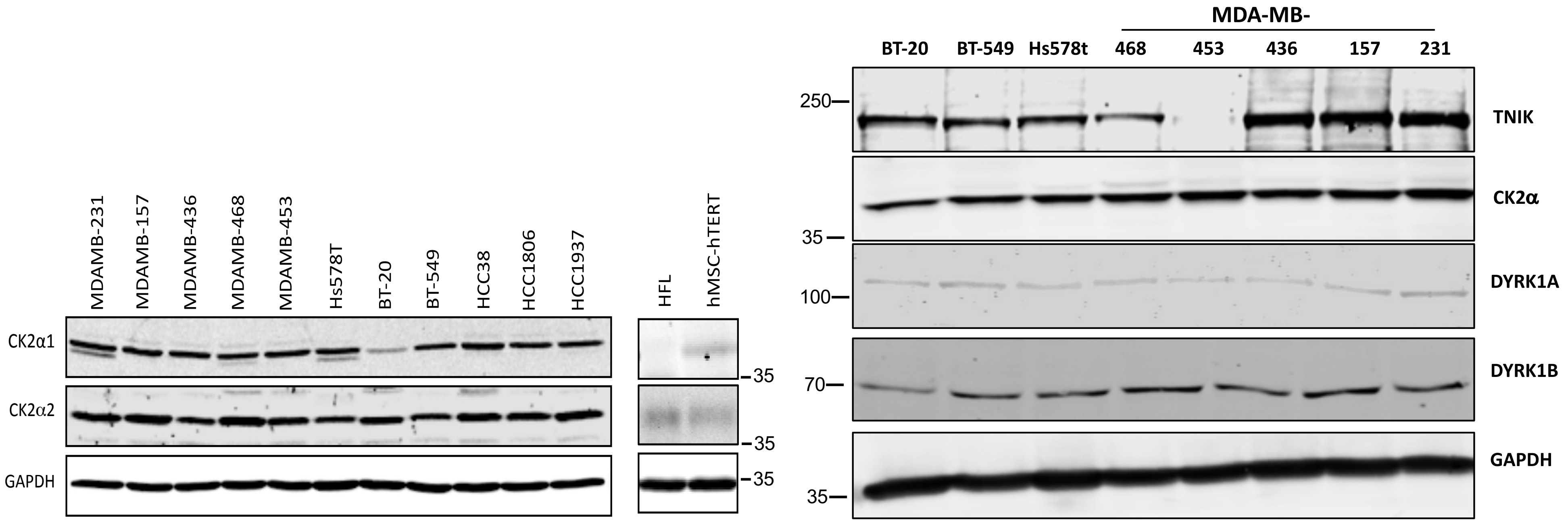

Supplementary Figure 4. Expression of 108600 target kinases in a panel of breast cancer and normal cell lines. Whole cell lysates derived from the indicated cell lines were resolved by SDS-PAGE and subjected to Western blot analysis using the antibodies directed against 108600 target kinases. GAPDH is shown as a loading control. Samples derive from the same experiment and the blots processed in parallel. Images are representative of 2 independent experiments. Source data are provided as a source data file.

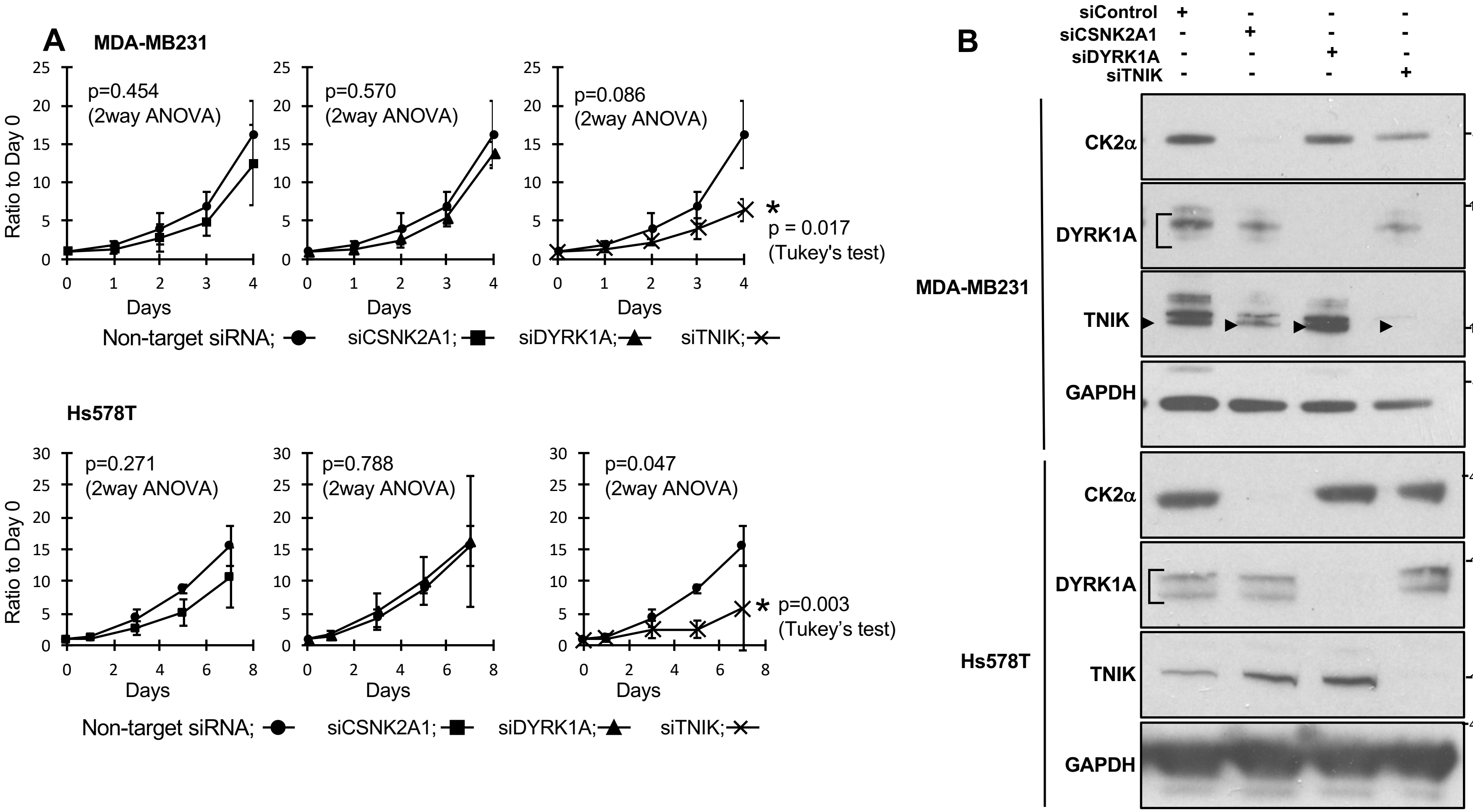

Supplementary Figure 5. Growth of MDA-MB231 and Hs578T cells transfected with siRNA pools individually targeting CK2, DYRK1, or TNIK. (A) MDA-MB231 and Hs578T cells were treated with siRNAs (30nM) that individually target 108600 substrates. Proliferation in monolayer cultures was determined by using trypan blue exclusion and manual counting. Mean value and standard deviation calculated from 3 independent experiments are plotted. Two-way ANOVA (two-sided) and Tukey's post-hoc tests (two-sided) were used to calculate the statistical difference between each treatment and for pairwise comparison of all data points, respectively. A p-value of less than 0.05 was regarded as statistically significant. (B) Downregulation of CK2, DYRK1 or TNIK expression in siRNA-transfected cells was confirmed by Western analysis 96 hours post-transfection. Results are representative of three independent experiments. Source data are provided as a source data file.

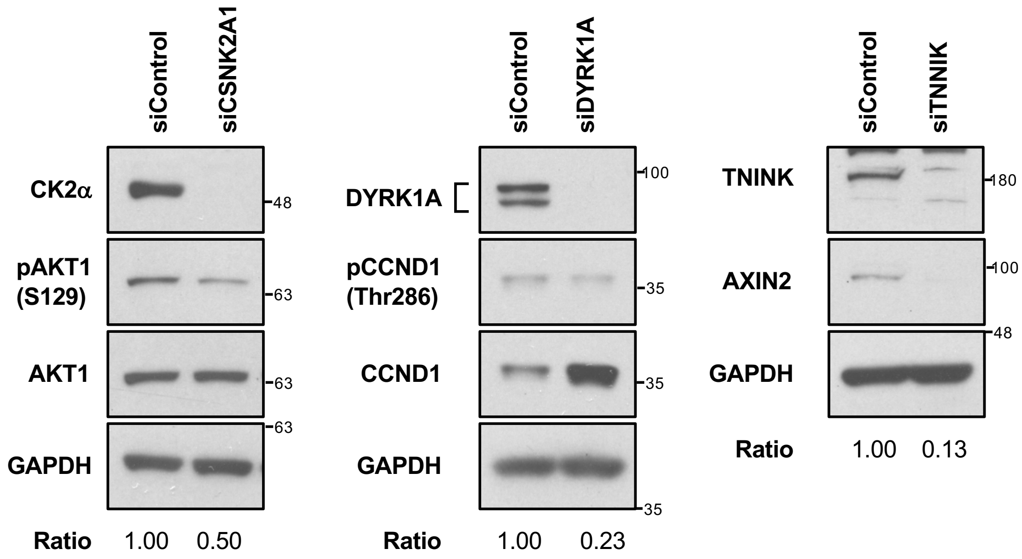

Supplementary Figure 6. siRNAs targeting CK2, DYRK1, or TNIK suppress their respective substrates and targets. MDA-MB-231 cells transfected with siRNAs (50nM) targeting CK2, DYRK1, or TNIK were lysed after 72 hours. Whole cell lysates were probed with the indicated antibodies. The numbers represent quantification of the relative levels of pAKT Ser129, pCCND1 Thr286 or AXIN2. Source data are provided as a source data file.

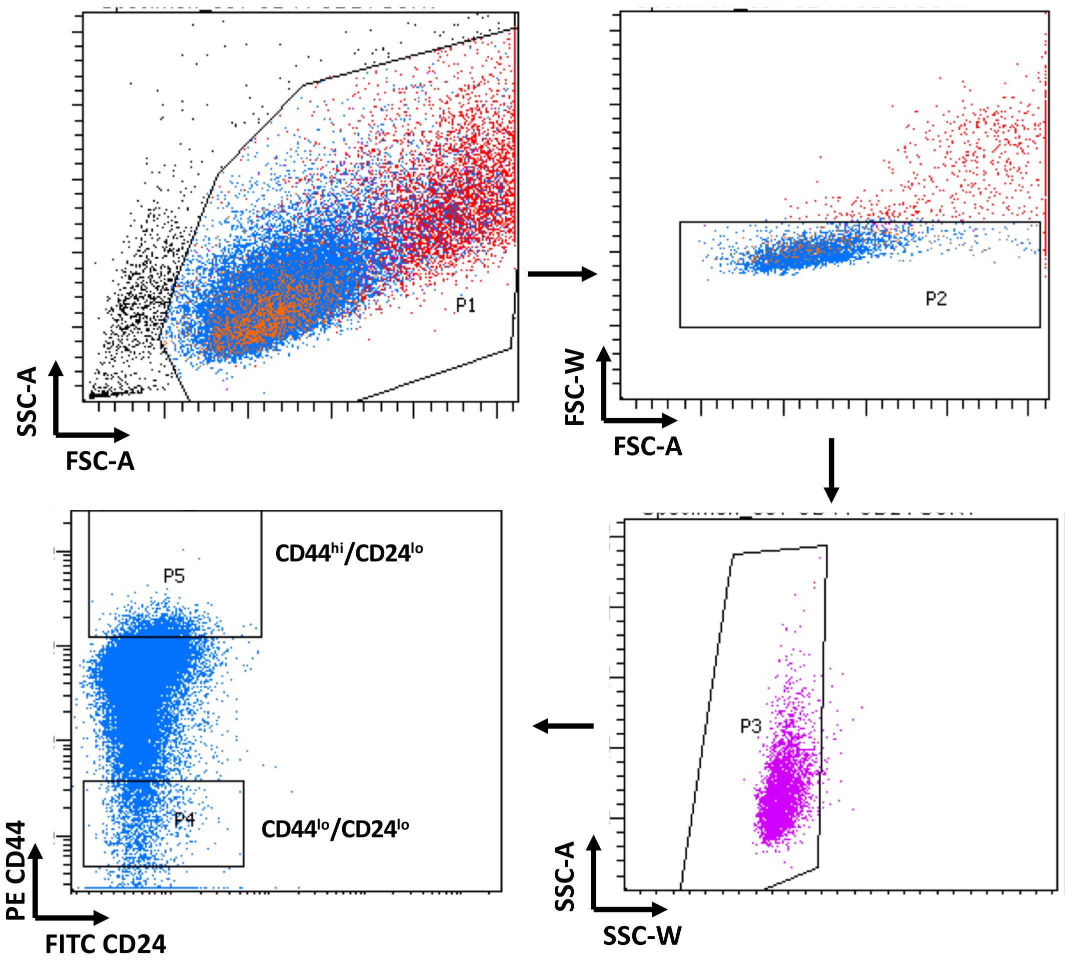

Supplementary Figure 7. Flow cytometry gating strategy used to isolate CD44^{high}h/CD24^{low} and CD44^{lo}/CD24^{lo} populations of MDA-MB-231 and Hs578T cells. The FACSDiva™-generated profile for MDA-MB-231 cells is shown and was used isolate the subsets analyzed in figure 1.

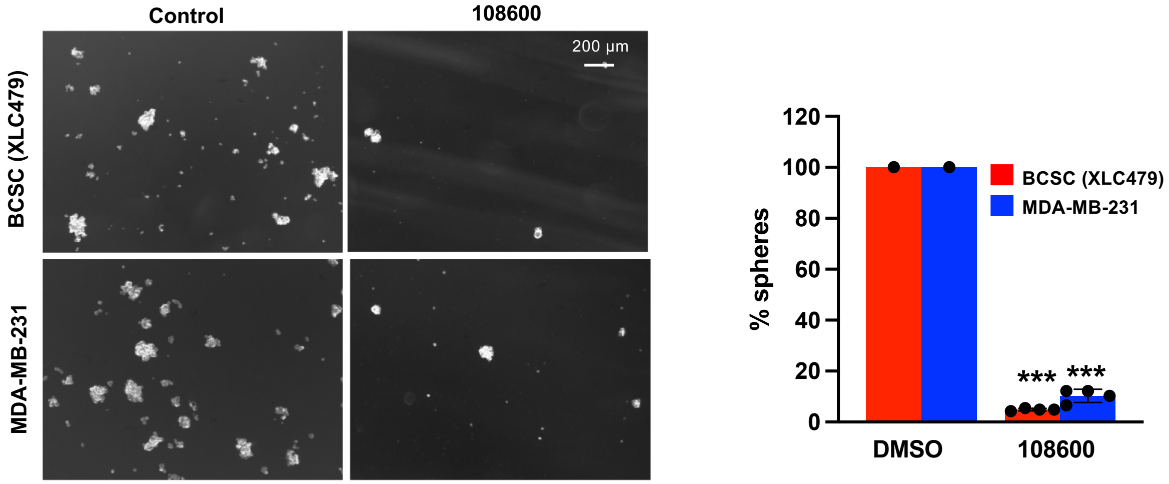

Supplementary Figure 8. Effect of 108600 on mammosphere forming ability of patient-derived breast cancer stem cells. CD44^{high} CD24^{low} cells isolated from a TNBC patient **(**XLC479, Creative Bioarray) were cultured in the presence and absence of 108600 (200nM) as described in the methods section. 108600-treated CD44^{high} CD24^{low} cells isolated from the MDA-MB-231 cell line are shown for comparison. Values represent the mean percentage of sphere formation $\pm$SD relative to that of DMSO, which was set to 100%, in 3 independent experiments. Statistical significance was determined using a t test (2-tailed). ***$p=1.36 \times 10^{-5}$ and 4.24×10^{-4} for 108600-treated CD44^{high} CD24^{low} TNBC patient BCSCs and MDA-MB-231 cells compared to their respective DMSO-treated controls. Source data are provided as a source data file.

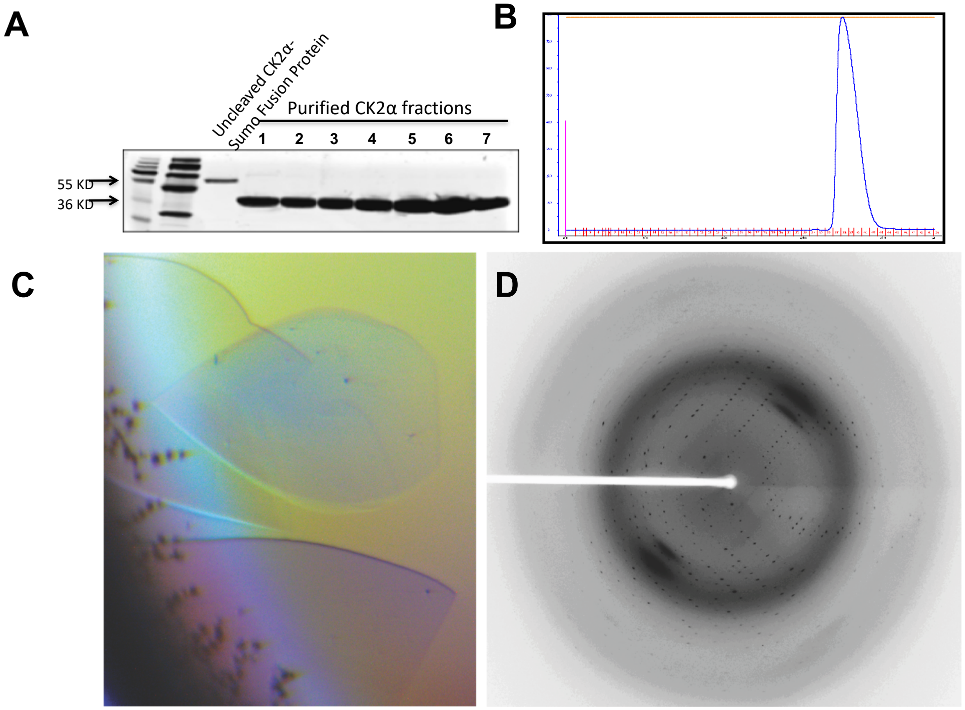

E

Data collection and refinement statistics

**Data collection**	
**Space group**	**$P2_1$**
**Cell dimensions:**	
a, b, c **(Å)**	**58.3, 45.6, 63.7**
**α, β, γ (°)**	**90.0, 111.8, 90.0**
**Complexes per AU[a]**	**1**
**Resolution range (Å)[b]**	**25-1.80 (1.84-1.80)**
**R_{merge} (%)**	**9.1 (50.0)**
**$I / \sigma I$**	**18.5 (2.1)**
**Completeness (%)**	**97.3 (77.7)**
**Redundancy**	**3.9 (2.8)**
**Refinement**	
**Resolution range (Å)**	**25-1.80**
**No. reflections**	**26,802**
**R_{factor}/R_{free}**	**17.5 /22.1**
**No. atoms**	
**Protein**	**2762**
**Ligand (108600)**	**34**
**Ions (SO_4^{2-})/ Ligand (other)**	**26**
**Water**	**222**
***B*-factors**	
**Protein**	**34.4**
**Ligand (108600)**	**39.2**
**Ions (SO_4^{2-})/ Ligand (other)**	**66.2**
**Water**	**41.7**
**R.m.s. deviations**	
**Bond length (Å)**	**0.005**
**Bond angles (°)**	**1.23**
**Ramachandran outliers[c]**	**0.3%**
**Sidechain outliers[c]**	**0.7%**
**Clashscore[c]**	**1**

[a] AU, asymmetric unit.
[b] Values in parentheses are for the highest-resolution shell.
[c] Percentage of Ramachandran and sidechain outliers as well as clashscore is calculated by the PDB X-ray Structure Validation Server.

Supplementary Figure 9. Purification and crystallization of CK2α. (A) A denaturing 4-20% SDS-polyacrylamide gel depicting the histidine-tag binding Ni-NTA chromatography step of purification of CK2α1 protein. The left lane on the gel shows the size marker with the arrows pointing to the 36 kDa and 55 kDa bands. The CK2α1 catalytic domain (residues 3-339, 44 kDa) was expressed in *E. coli* as a His6-SUMO (small ubiquitin-like modifier) N-terminally tagged fusion protein (molecular weight 56 KDa). The second lane from the left of the gel shows the cell lysate before loading onto the Ni-NTA column. The third lane of the gel shows the His6-Sumo-CK2α1 protein after the first run on the Ni-NTA column. The His6-SUMO tag was removed by cleavage with Ulp1 protease and the protein was loaded on the Ni-NTA column second time and collected in flow-through in seven fractions (lanes 4-10 on the gel). CK2α1 protein was further purified using a heparin column. A single, large scale protein preparation was performed to obtain sufficient quantities of protein for subsequent crystallography and enzymatic assays. (B) Final step of CK2α1 protein purification by size-exclusion gel-filtration column chromatography. The protein was loaded onto a Superdex 75 column and the chromatography trace is shown at 280 nm wavelength. (C) Crystals of CK2α1-108600 complex obtained by the vapor diffusion method at 20°C. (D) The diffraction of CK2α1-108600 complex crystals to 1.80 Å resolution at X25 synchrotron beamline at Brookhaven National Laboratory. (E) Data collection and refinement statistics for the CK2α1-108600 complex structure.

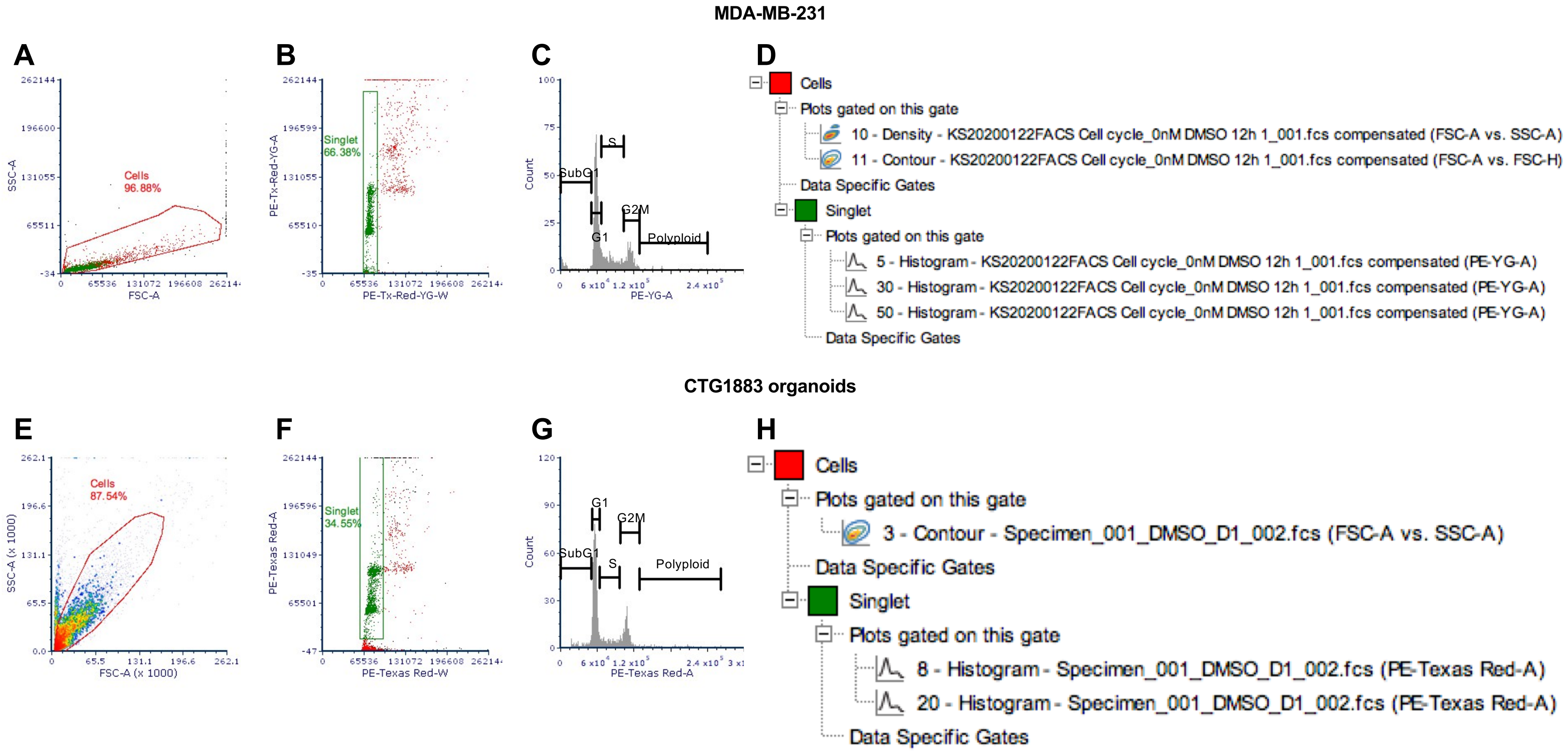

Supplementary Figure 10. Gating strategy for cell cycle progression analysis. Cell size and granularity (A, E), singlets (B, F) and each phase of the cell cycle (C, G) were analyzed in MDA-MB-231 cells (A-D) and CTG1883 organoids (D-E). The gating hierarchy for MDA-MB-231 and CTG1883 organoids are shown in panels D and H, respectively. This gating strategy was used to analyze the cell cycle progression in cell lines and organoids shown in figure 4 and supplementary figure 13.

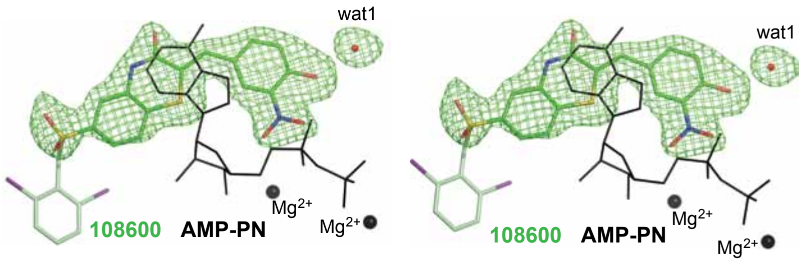

Supplementary Figure 11. Stereo view of an electron density map for the ligand in the active site of CK2α1. A simulated annealing Fo-Fc omit electron density map for a ligand in the CK2α1-108600 structure is contoured at 3.0 σ-level at 1.80 Å resolution and is colored in green. 108600 fits the map and is shown in sticks with its carbon atoms colored in green, oxygen atoms in red, nitrogen atoms in blue and sulfur atoms in yellow. The water molecule (wat1) is shown as a red sphere. The electron density for the 2,6-dichlorobenzyl ring of the drug is disordered due to the conformational flexibility of this aromatic moiety in the solvent-exposed region of the active site. Shown in black lines is AMP-PNP (PDB ID: 3NSZ) 21 in the active site of CK2α1. The CK2α1-108600 and CK2α1-AMP-PNP structures are superimposed by the main chain of the protein. The γ-phosphate of AMP-PNP is hydrolyzed in 3NSZ to produce AMP-PN due to the acidic crystallization conditions. The α- and β-phosphate groups of the AMP-PNP coordinate two magnesium ions shown as black spheres. The AMP-PN-2Mg2+ does not fit the electron density map.

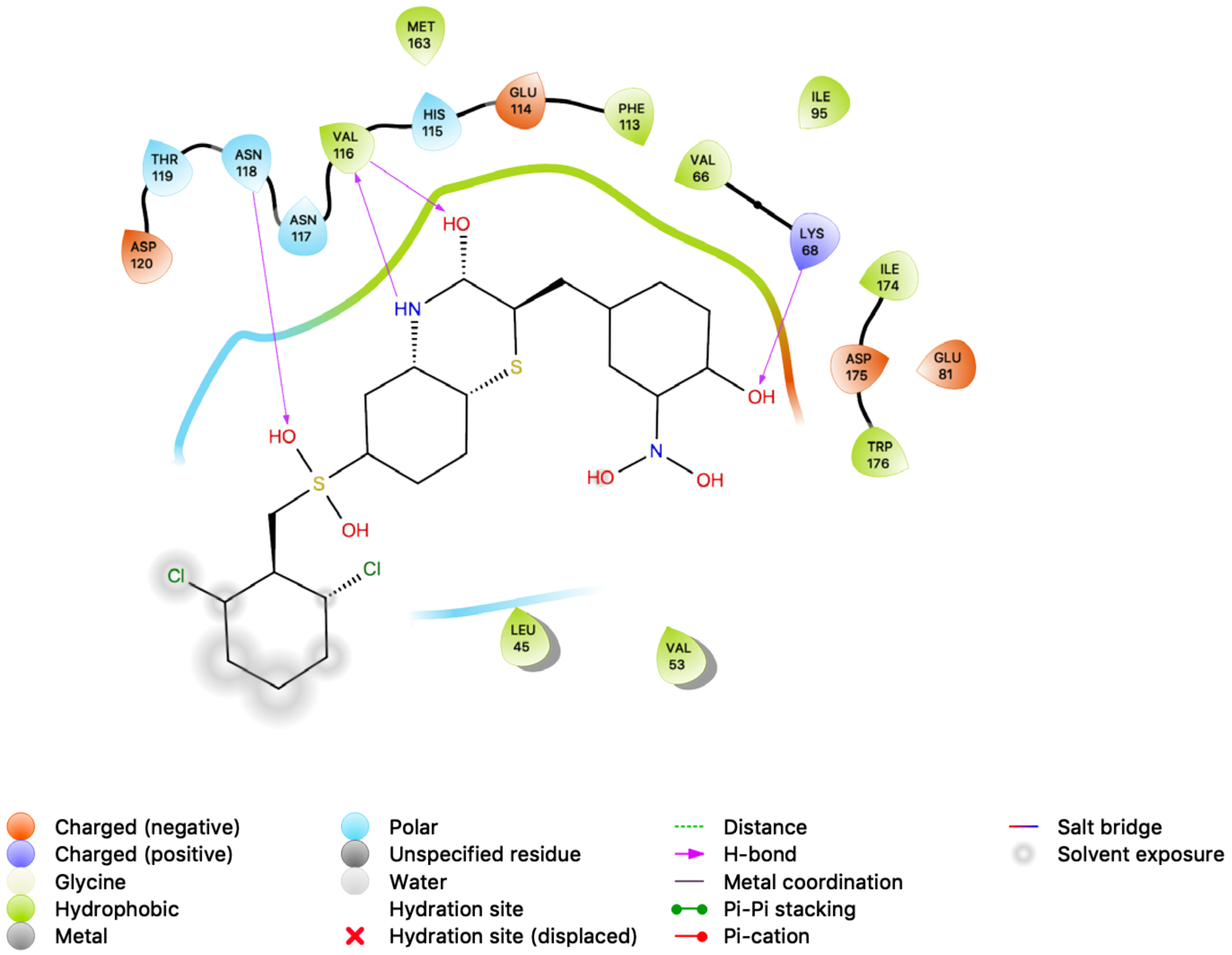

Supplementary Figure 12. Ligand interaction diagram for 108600 in the active site of the CK2α1 protein. The diagram was produced with the Maestro module in the Schrodinger suite of programs (Schrodinger LLC).

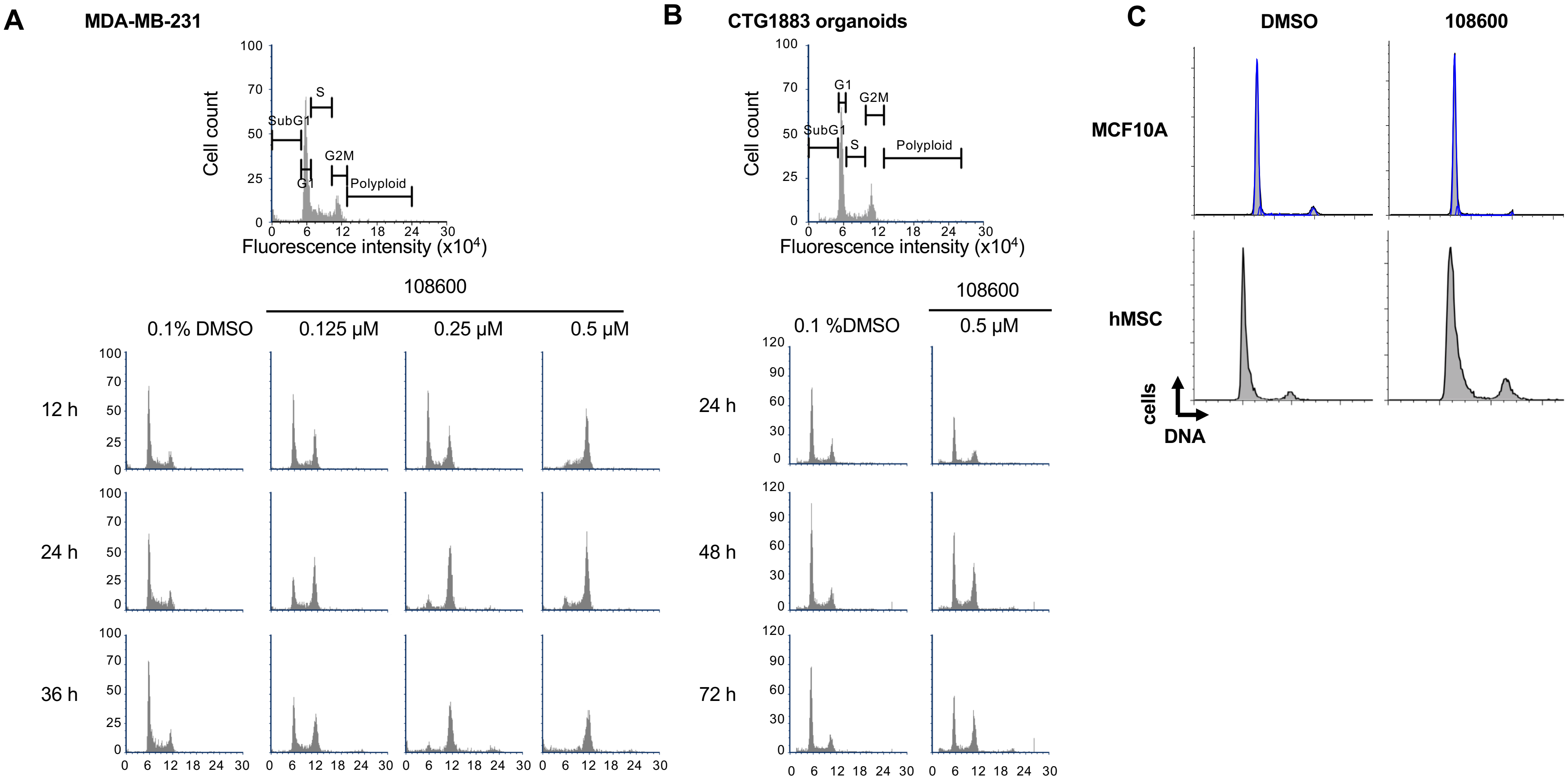

Supplementary Figure 13. 108600 induces G2/M arrest and apoptosis of TNBC cells and organoids. MDA-MB-231 cells (A) and CTG1883 organoids (B) were treated with the indicated concentrations of 108600 for 12, 24 and 36 hrs, stained with propidium iodide (PI) and subjected to flow cytometric analysis. Gating strategies are shown. (C) Effect of 108600 on cell cycle progression of MCF-10A and human mammary stem cells (hMSC). Cells were treated with 1μM 108600 for a 24-hour period, stained with PI and subjected to flow cytometric analysis.

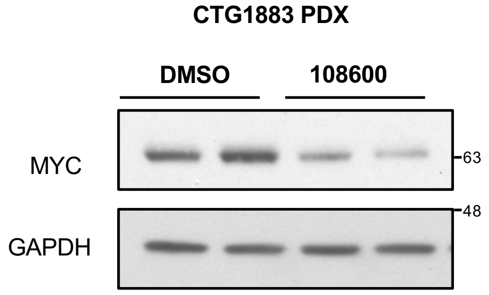

Supplementary Figure 14. Treatment with 108600 decreases c-MYC expression in CTG1883 tumors. CTG1883 tumor-bearing NSG mice were treated with 100 mg/kg 108600 (n=2). The tumors were harvested 3 hours after the second injection and immediately snap frozen in liquid nitrogen. Whole cell extracts were prepared as described in the methods section and subjected to Western blot analysis using MYC and GAPDH-specific antisera. Whole cell extracts were prepared from 2 individual tumor bearing mice simultaneously and subjected to Western blot analysis. Source data are provided as a source data file.

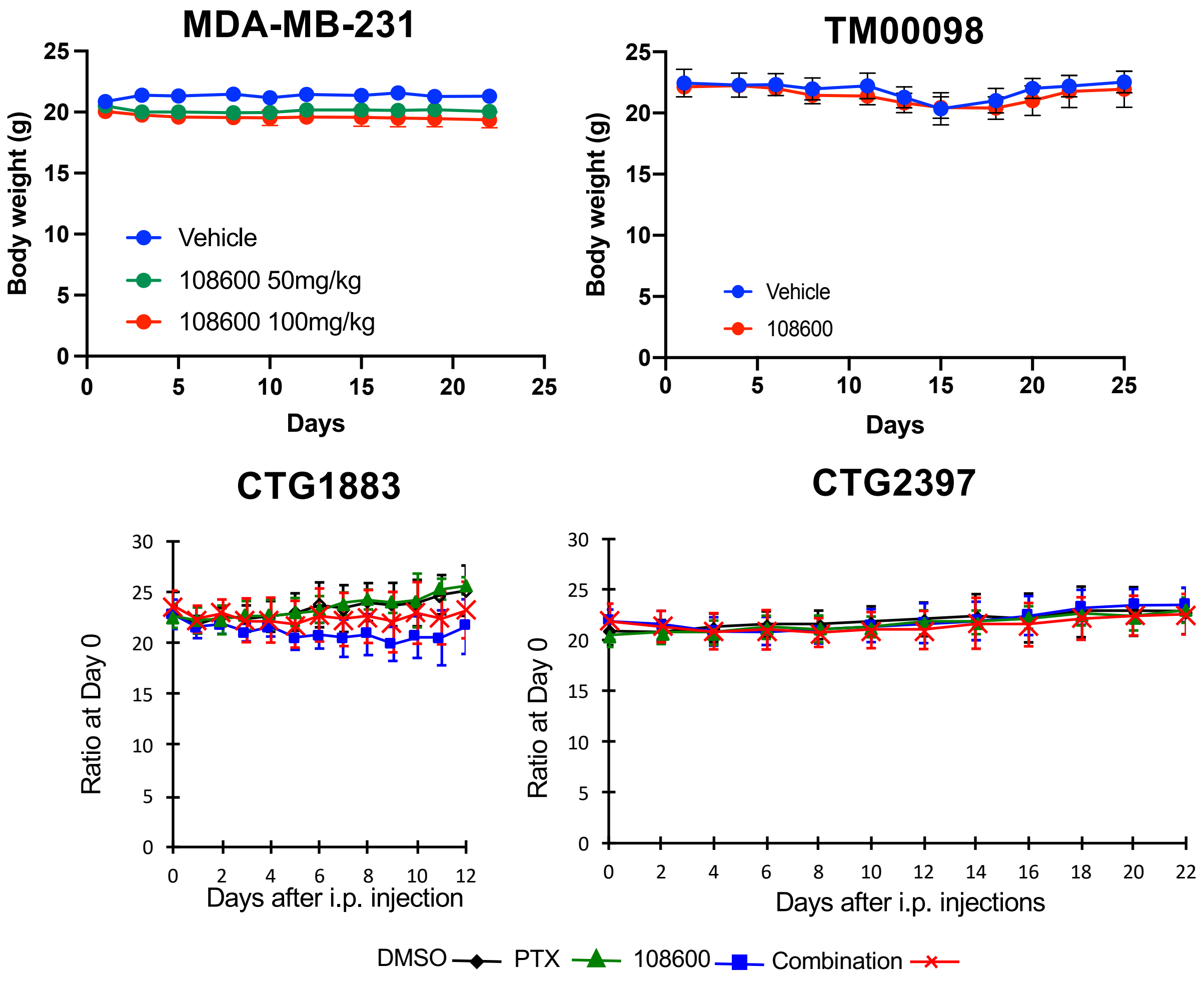

Supplementary Figure 15. Body weights of tumor-bearing mice treated with 108600. The body weights of vehicle and 108600-treated mice were determined on the indicated days and plotted as a function of time as outlined in figure 6. Statistical differences in body weights among the different treatment groups were determined using 2-way ANOVA. All data are presented a mean ± standard deviation. Source data are provided as a source data file.

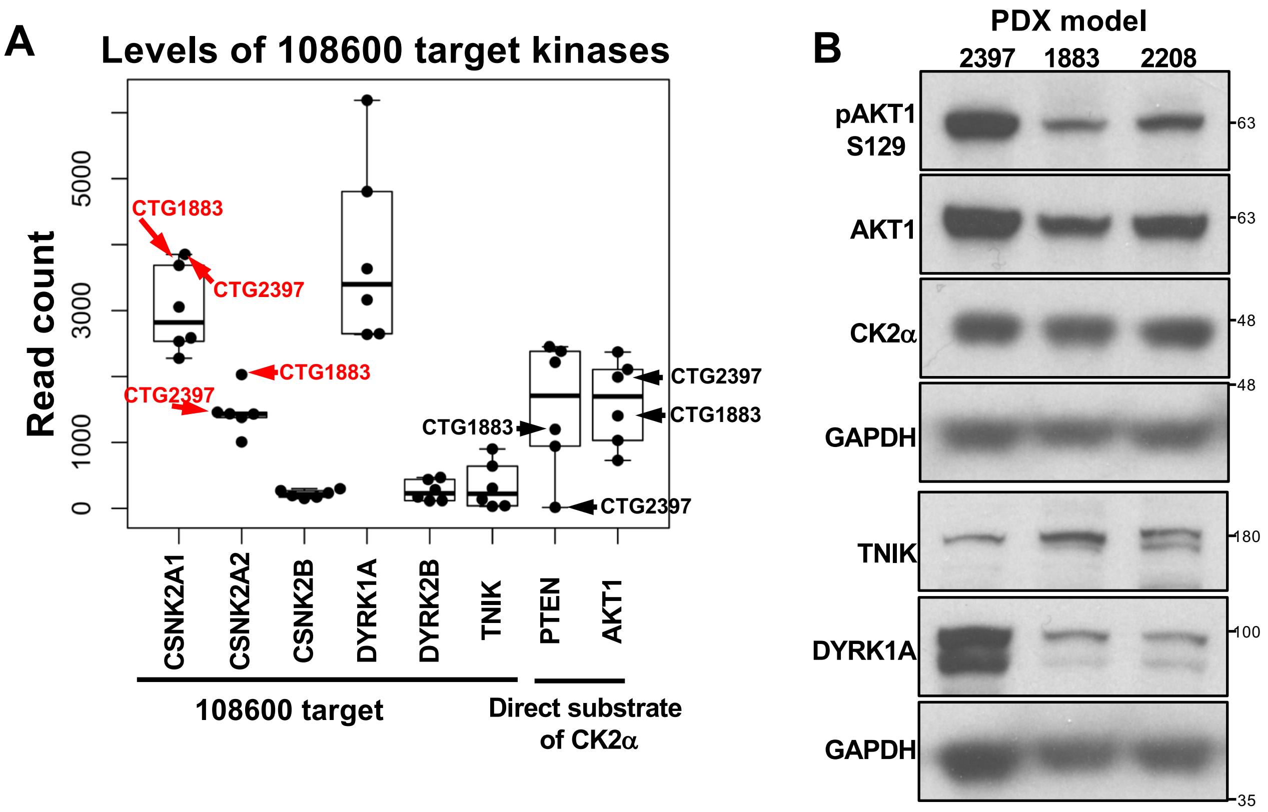

Supplementary Figure 16. Expression of 108600 targets in PDX models (A) Transcript levels of 108600 targets in TNBC PDX tumorgraft models. The expression of 108600 targets were assessed by analyzing the read count of high throughput RNA sequencing. The output FASTQ files were mapped by STAR (version 2.4.0c) into reference genome (GRCh37.p12, Genecode release 18) and the reads were quantified by featureCounts (v1.4.3-p1). The output read counts, which was normalized by the Relative Log Expression method using DEseq2 (Bioconductor), were used to determine the expression of CSNK2A1 (ENSG00000101266.12), CSNK2A2 (ENSG00000070770.4), DYRK1A (ENSG00000157540.15), DYRK1B (ENSG00000105204.9), TNIK (ENSG00000154310.12), PTEN (ENSG00000171862.5) and AKT1 (ENSG00000142208.11). Each data point represents data obtained for an individual animal, with the top and bottom of the box corresponding to the 75 and 25 percentiles, respectively. Data points that are higher or lower than 1.5 times that of the interquartile range within the box were regarded as outliers. (B) Expression of 108600 target proteins in TNBC PDX models. Portions of cryopreserved primary tumors expressing high levels of 108600 targets at the RNA level were subjected to a single Western blot analysis using the indicated antibodies. CTG1883 and CTG2397 were chosen for expansion and subsequent efficacy studies based on the expression of 108600 target kinases at the protein level.

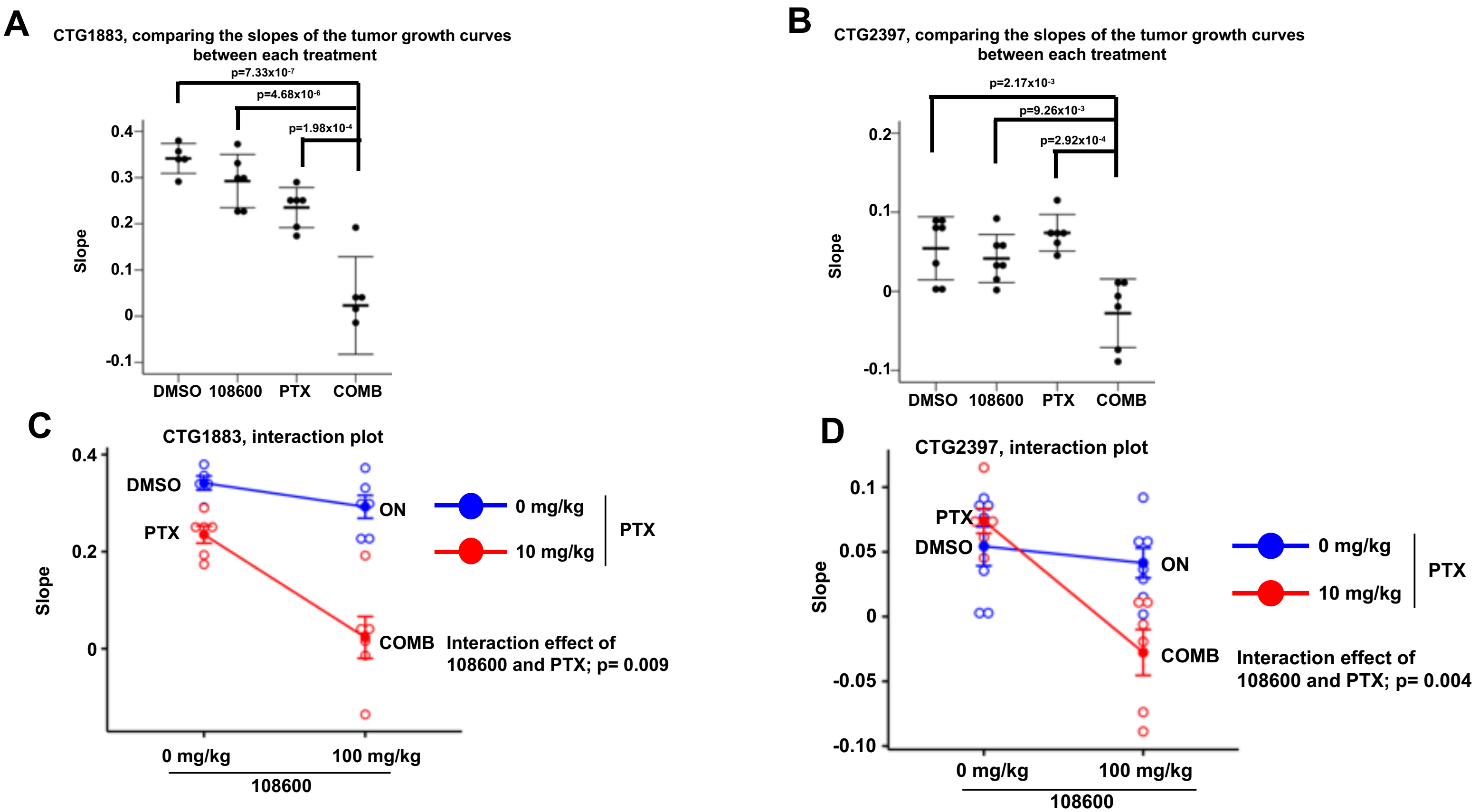

Supplementary Figure 17. Synergistic inhibition by 108600 and paclitaxel on tumor growth of the CTG1883 and CTG2397 PDX models. The tumor volumes shown in figure 6E were normalized using Log2 transformation and subjected to linear regression analysis in order to calculate the slopes of the growth curves. (A) The slope of the tumor growth curve for the CTG1883 tumor. (B) The slope of the tumor growth curve for the CTG2397 tumor. Data points are representative of individual animals. The bold horizontal line and error bars represent mean ± standard deviation. The slopes for each treatment were compared using Tukey's test (two-sided), and p-values of less than 0.05 were regarded as statistically significant. (C) Interaction effect between 108600 and paclitaxel (PTX) in the CTG1883 tumor. (D) Interaction effect between 108600 and PTX in the CTG2397 tumor. The interaction effect and statistical significance for (C) and (D) were calculated by 2-way ANOVA (two-sided). A p-value of less than 0.05 is regarded as statistically significant. As a statistically significant difference was detected between blue and red lines for both tumors, the effects of 108600 and PTX combination treatment on tumor growth are synergistic. The filled points and error bars represent mean ± standard deviation. Open circles represent values for individual mice.

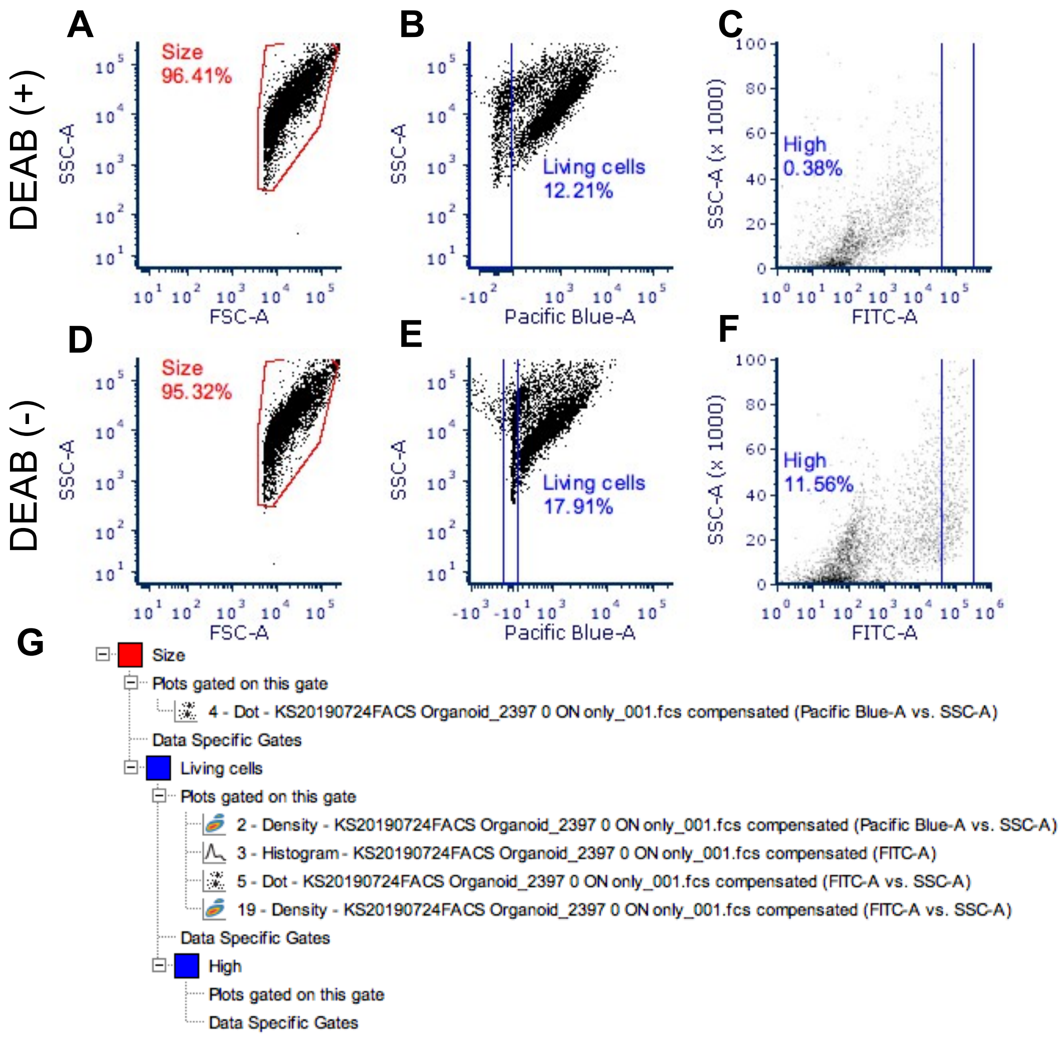

Supplementary Figure 18. Gating strategy used in the Aldeflour assay. Viable cells with similar size and granularity (A, B, D and E) were selected and the frequencies of Aldeflour high cells were measured by comparison with DEAB treated cells (C, F). The gating hierarchy is shown in (G). This gating strategy was used to analyze the frequency of ALDH^{high} cells presented in figure 6.
