# Supplementary material for: Simultaneous CK2/TNIK/DYRK1 inhibition by 108600 suppresses triple negative breast cancer stem cells and chemotherapy-resistant disease
Source: Nat Commun. 2021 Aug 3;12:4671. doi: 10.1038/s41467-021-24878-z (PMC8333338; doi:10.1038/s41467-021-24878-z)
Supplement: Supplementary file 2 — Reporting Summary [file 41467_2021_24878_MOESM2_ESM.pdf]

nature research

Corresponding author(s): E. Premkumar Reddy, Ph.D., Hanna Y. Irie, MD., Ph.D.

Last updated by author(s): June 30, 2021

Reporting Summary

Nature Research wishes to improve the reproducibility of the work that we publish. This form provides structure for consistency and transparency in reporting. For further information on Nature Research policies, see our Editorial Policies and the Editorial Policy Checklist.

Statistics

For all statistical analyses, confirm that the following items are present in the figure legend, table legend, main text, or Methods section.

n/a	Confirmed	
☐	☒	The exact sample size (*n*) for each experimental group/condition, given as a discrete number and unit of measurement
☐	☒	A statement on whether measurements were taken from distinct samples or whether the same sample was measured repeatedly
☐	☒	The statistical test(s) used AND whether they are one- or two-sided<br>*Only common tests should be described solely by name; describe more complex techniques in the Methods section.*
☐	☒	A description of all covariates tested
☐	☒	A description of any assumptions or corrections, such as tests of normality and adjustment for multiple comparisons
☐	☒	A full description of the statistical parameters including central tendency (e.g. means) or other basic estimates (e.g. regression coefficient) AND variation (e.g. standard deviation) or associated estimates of uncertainty (e.g. confidence intervals)
☐	☒	For null hypothesis testing, the test statistic (e.g. *F*, *t*, *r*) with confidence intervals, effect sizes, degrees of freedom and *P* value noted<br>*Give P values as exact values whenever suitable.*
☒	☐	For Bayesian analysis, information on the choice of priors and Markov chain Monte Carlo settings
☒	☐	For hierarchical and complex designs, identification of the appropriate level for tests and full reporting of outcomes
☒	☐	Estimates of effect sizes (e.g. Cohen's *d*, Pearson's *r*), indicating how they were calculated

Our web collection on statistics for biologists contains articles on many of the points above.

Software and code

Policy information about availability of computer code

Data collection	Flow cytometric data was acquired using FACSDiva v8.0. Bioluminescence imaging data was acquired using Living Image v4.7.2.
Data analysis	Flow cytometric data was analyzed using Flowjo v9.0 or FCS Express v6.0. Bioluminescence data was analyzed using Living Image v4.7.2. Statistical analysis was performed using Prism v9.0. Microscopy data was analyzed using Image J v1.5.

For manuscripts utilizing custom algorithms or software that are central to the research but not yet described in published literature, software must be made available to editors and reviewers. We strongly encourage code deposition in a community repository (e.g. GitHub). See the Nature Research guidelines for submitting code & software for further information.

Data

Policy information about availability of data

All manuscripts must include a data availability statement. This statement should provide the following information, where applicable:

- Accession codes, unique identifiers, or web links for publicly available datasets
- A list of figures that have associated raw data
- A description of any restrictions on data availability

Atomic coordinates and structure factors have been deposited in the Protein Data Bank (PDB) under accession code 7L1X: https://www.rcsb.org/structure/unreleased/7L1X

Field-specific reporting

Life sciences study design

All studies must disclose on these points even when the disclosure is negative.

Sample size	The sample sizes for the in vivo mouse studies were determined based on initial pilot studies performed with each strain of mice and effect sizes estimated from these studies. The final sample sizes were based on power calculations (two sample t-test) with power >0.85
Data exclusions	No data was excluded.
Replication	Replicate studies were performed as indicated in the figure legends of the main manuscript and supplemental text.
Randomization	Mice with equivalent tumor burdens were randomized to individual treatment groups.
Blinding	The treatment groups in all xenograft efficacy studies were not blinded in order to avoid errors in **that might have resulted in animals being given the wrong treatment after they were assigned to groups.**

Reporting for specific materials, systems and methods

We require information from authors about some types of materials, experimental systems and methods used in many studies. Here, indicate whether each material, system or method listed is relevant to your study. If you are not sure if a list item applies to your research, read the appropriate section before selecting a response.

Materials & experimental systems

n/a	Involved in the study	
☐	☒	Antibodies
☐	☒	Eukaryotic cell lines
☒	☐	Palaeontology and archaeology
☐	☒	Animals and other organisms
☐	☒	Human research participants
☒	☐	Clinical data
☒	☐	Dual use research of concern

Methods

n/a	Involved in the study	
☒	☐	ChIP-seq
☐	☒	Flow cytometry
☒	☐	MRI-based neuroimaging

Antibodies

Antibodies used	Primary antibodies were purchased from Cell Signaling Technologies: phospho-AKT1 (Ser129) (#13461), DYRK1A (#2771), DYRK1B (#2703), AKT1 (#2967), phospho-CYCLIN D1 (Thr286) (#3300), CYCLIN D1 (#2926), p27 (#3698), AXIN2 (#2151), TNIK (#32712), cleaved PARP (#9541 and #32563), PARP (#9532 and #9542), cleaved Caspase 3 (#9661), CD44 (#3507), MDR-1 (#13342), c-Myc (#5605); Abcam: CK2 alpha1 (ab76040), phospho-p27 (Ser10) (ab62364), pericentrin (ab4448); Santa Cruz Biotechnology: GAPDH (sc-365062), TNIK (sc-136103), CK2 alpha 2 (sc-9030), EGFR (sc-53274); BD Biosciences: PE CD44 (561858, 1:500), FITC CD24 (560992, 1:200); Invitrogen: beta-catenin (AH00462); phospho-DYRK (PA5-64574). Secondary antibodies were purchased from Cell Signaling Technologies (horse radish peroxidase [HRP] conjugated anti-rabbit IgG [#7074]) or LICOR Biosciences (IRDye® 680 Goat anti-Mouse IgG (H + L) #926-32220, IRDye® 800CW Goat anti-Rabbit IgG (H + L), #926-32211, IRDye® 680RD Goat anti-Mouse IgG #926-68070, IRDye® 680RD Goat anti-Mouse IgG #926-32210). Primary and secondary antibodies for Western blot analysis were used at 1:1,000 and 1:10,000 dilutions, respectively. Antibodies were used at a dilution of 1:200 in immunofluorescence staining.
Validation	**All primary a**ntibodies were previously validated by the manufacturers, as stated on their websites. Cell signaling technologies validates its phospho-specifc antibodies using either stimulated or phosphatase-treated samples. Antibodies directed against pan proteins are tested in multiple cell lines that express varying levels of protein across different species. Santa Cruz validates antibodies directed against pan proteins using multiple cell lines across different species. BD Biosciences tests its antibodies on "primary cells, cell lines and/or transfectant cell models with relevant controls using multiple immunoassays to ensure biological accuracy." Antibodies purchased from Abcam are knock-out validated or validated in cells with established levels of protein expression. Antibodies used in IF are validated by "looking at cells that either do or do not express the target protein within the same tissue." Antibodies purchased from Invitrogen and Sigma Aldrich are validated using methods identical to those of CST and Santa Cruz Biotechnology.

Eukaryotic cell lines

Policy information about cell lines

Cell line source(s)	ATCC: MDA-MB-231, MDA-MB-157, MDA-MB-436, MDA-MB-468, MDA-MB-453, BT-20, Hs578T, HCC1806, MCF-7, BT-474, MCF-10A, HFL, hMSC-hTERT.
Authentication	Authentication of cell lines was performed by ATCC prior to purchase by comparing the STR profile of sample cell lines with its human Cell STR database. Cell lines were not independently re-authenticated.
Mycoplasma contamination	All cell lines are negative for mycoplasma contamination.
Commonly misidentified lines (See ICLAC register)	none used

Animals and other organisms

Policy information about studies involving animals; ARRIVE guidelines recommended for reporting animal research

Laboratory animals	Organism: Mus musculus. Strains: NCr-nu/nu, Prkdcscidll2rgtm1Wjl/SzJ (NSG). Commercially available PDX models: TM00098 PDX-bearing NSG mice. All mice were female and were 8-12 weeks of age at the start of all studies. Animals were housed in climate-controlled barrier facilities with automated light/dark cycles.
Wild animals	Study does not involve wild animals

Field-collected samples	Study does not involve field-collected samples
Ethics oversight	All animal experiments were performed under protocols approved by the Icahn School of Medicine at Mount Sinai's Institutional Animal Care and Use Committee according to federal and institutional guidelines and regulations.

Note that full information on the approval of the study protocol must also be provided in the manuscript.

Human research participants

Policy information about studies involving human research participants

Population characteristics	Patient derived xenograft models were generated from tumor tissue from consented adult patients diagnosed with stage 1-3 triple negative breast cancer (male and female, all ethnic/racial groups). Covariate-relevant age characteristic of the population-age: the mean age of the consented patients was 49. This is on par with the average age of diagnosis reported for TNBC which is diagnosed at a younger age than other subtypes.
Recruitment	Samples from patients treated at the Mount Sinai Hospital were collected for PDX generation and expansion. Self selection bias: Consented patients sought and received breast cancer care at a tertiary level cancer center. Nevertheless, the response rate of patient cancers to chemotherapy is comparable to the average published rates for this subtype such that the range of inherent biological characteristics related to chemotherapy sensitivity was maintained.
Ethics oversight	Written informed consent was obtained for all subjects on Icahn School of Medicine at Mount Sinai (ISMMS) Institutional Review Board (IRB)–approved protocol (HSM# 14-00330) prior to the procedure at which the specimen was obtained. The studies were conducted in accordance with the Belmont Report and U.S. Common Rule and approved by the ISMMS IRB.

Note that full information on the approval of the study protocol must also be provided in the manuscript.

Flow Cytometry

Plots

Confirm that:

- [x] The axis labels state the marker and fluorochrome used (e.g. CD4-FITC).
- [x] The axis scales are clearly visible. Include numbers along axes only for bottom left plot of group (a 'group' is an analysis of identical markers).
- [x] All plots are contour plots with outliers or pseudocolor plots.
- [x] A numerical value for number of cells or percentage (with statistics) is provided.

Methodology

Sample preparation	For sorting, single cell suspensions were prepared using PBS supplemented with 2% heat inactivated FBS
Instrument	LSRII Fortessa (analysis), Arial II (sorting), BD Biosciences.
Software	Data for analytical purposes were analyzed using FCS Express 6.0. Quantitation/re-analysis of sorted populations was performed using Diva 8.0
Cell population abundance	Sorted populations were re-analyzed post-sort on a Arial II to confirm purity.
Gating strategy	Single cells were gated for sort purification and/or analysis using the following strategy: 1) FSC vs SSC to gate out dead cells and debris. 2) SSC-A vs SSC-H to select single cells. 3) FSC-A vs FSC-H to further confirm that all cells analyzed were single cells. The plots used to quantitate the indicated populations are shown in the figures of the main manuscript and supplemental file.

- [x] Tick this box to confirm that a figure exemplifying the gating strategy is provided in the Supplementary Information.
